# Obscurin a localizes near the cell membrane to modulate stress fiber dynamics and cell migration

**DOI:** 10.1016/j.jbc.2026.111383

**Published:** 2026-03-17

**Authors:** Kamrin D. Shultz, Stephanie N. Ouderkirk, Yasmin F. Al Anbari, C. Jackson White, Peter Henry, Kristopher E. Kubow, Daniel E. Conway, Callie J. Miller, Nathan T. Wright

**Affiliations:** 1 Department of Chemistry & Biochemistry, James Madison University, Harrisonburg, Virginia, USA; 2 Department of Biology, James Madison University, Harrisonburg, Virginia, USA; 3 Department of Biomedical Engineering, Ohio State University, Columubus, Ohio, USA; 4 Department of Engineering, James Madison University, Harrisonburg, Virginia, USA

**Keywords:** actin, cancer, cell migration, confocal microscopy, EMT, metastasis, obscurin, PIP_3_

## Abstract

Obscurin is a giant (720–900 kDa) modular cytoskeletal protein with multiple signaling domains. While it is most highly expressed in myocytes, obscurin is also the second most mutated protein in breast and colorectal cancers and is significantly downregulated in pancreatic cancer. Obscurin derives its antioncogenic properties, at least in part, through its ability to modulate cellular motility and migration; obscurin knockdown in cultured epithelial cells leads to increased migration and an epithelial-to-mesenchymal transition (EMT). Obscurin likely controls cell motility through the obscurin RhoGEF domain interaction with the RhoA/ROCK pathway and/or the obscurin PH domain interaction with a PI3K/PIP3 pathway. Here, we more fully describe which obscurin domains dictate subcellular localization and regulate cellular motility. The obscurin C-terminus adenovirally infected into MDCK and MCF - 10A cells localizes to adhesion structures at the plasma membrane. This localization is driven by four regions in obscurin: the obscurin RhoGEF and PH domains, along with two regions in the unstructured C-terminus. Infected cells lack central stress fibers, and this morphology is linked to the RhoGEF domain, the PH domain, and the C-terminal 76 residues. These three obscurin regions also inhibit cell motility. Together these data demonstrate how both specific obscurin domains and specific cellular localization regulate cellular velocity.

To initiate tissue regeneration, wound healing, inflammation, and a host of other physiological processes, epithelial cells convert from tightly-interacting, fully differentiated, stationary cells into looser interacting, less differentiated, motile mesenchymal cells (1, 2, 3). When dysregulated, this process, termed epithelial-to-mesenchymal transition (EMT), is one hallmark in epithelial cancer progression (4). EMT is controlled by multiple independent molecular pathways. Prominent EMT signaling axes include mechanisms that control cadherin signaling (*e.g.* the Notch and Wnt signaling pathways), mechanisms that rearrange the cytoskeleton (*e.g.* the integrin/SOS signaling pathway), and mechanisms that alter focal adhesion and stress fiber formation (*e.g.* the phosphoinositide-3-kinase (PI3K) and RhoA signaling pathways) (5, 6, 7, 8).

The biochemical pathways regulating EMT cell motility are under strict spatial control. For instance, moderate RhoA activation near the leading edge of a migrating cell stimulates actin polymerization, actin branching, and lamellipodia formation, while higher RhoA levels at the trailing edge stimulate stress fiber formation and cell retraction (9, 10, 11, 12, 13). As another example, PI3K produces extra phosphatidylinositol (3, 4, 5)-trisphosphate (PIP_3_) at the leading edge of motile cells, which activates the mTORC complexes and results in increased actin polymerization (14, 15, 16, 17, 18). This, in turn, stimulates filopodia extension and increases cell migration in epithelia, but only in the vicinity of the increased PIP_3_ levels (19). Thus, there is a need to know both the molecular mechanism and the subcellular location of motility modulators in order to fully describe how cells control motility, and how cancers hijack this control.

Obscurin is a large (720–900 kDa) cytoskeletal protein composed primarily of a series of more than 60 modular domains (Fig. 1) (20, 21, 22). Obscurin expression is highest in striated muscle and was initially described as a regulator of muscle cell architecture and calcium handling, as it is the only known protein that directly binds to both the contractile apparatus and the surrounding sarcoplasmic membrane structure (23, 24, 25, 26). In muscle cells, the N-terminus binds to titin, myomesin, and MyBP-C, while the C-terminus binds ankyrin (9, 22, 27, 28, 29). All of the known obscurin signaling domains are near the C-terminus; obscurin contains a calmodulin-binding IQ domain, a RhoGEF domain, and a PH domain (30). The obscurin-B isoform also contains two kinase domains that can phosphorylate N-cadherin (31, 32).

**Figure 1:**
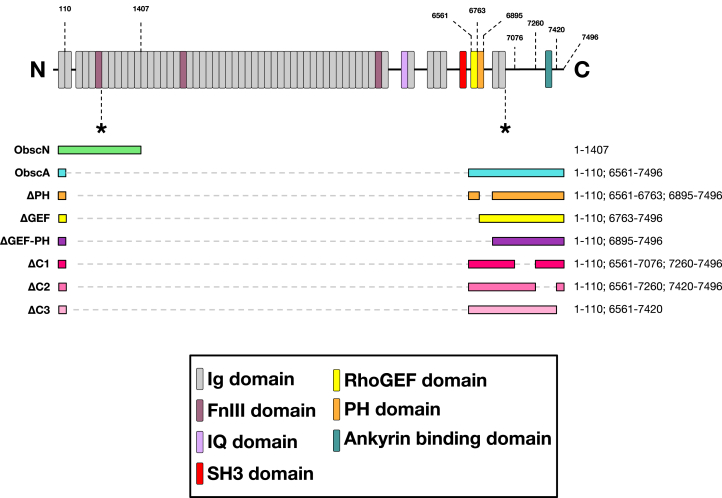
**Obscurin is near cell membrane proteins.***Top*, Obscurin domain architecture, with the adenovirally-expressed constructs below. The × symbols denote location of either the BirA biotin ligase or the mTFP fluorescent insert. *Bottom*, selected BioID Assay results, showing those proteins most often in biotinylated in the presence of ObscA-BirA, as compared to a sham-infected control.

A 2006 metagenomic study identified the gene encoding obscurin as the second most mutated gene in breast and colorectal cancers, after *P53*, with many of the documented mutations resulting in premature stop codons throughout the length of obscurin (33). Further studies confirmed that both obscurin A and B, along with a complex assortment of shorter isoforms, are expressed at low levels across a variety of epithelial-derived tissues (34, 35). While the precise isoform distribution is still being explored, the mere existence of non-muscle obscurin that correlates with cancer has led to a growing appreciation of obscurin’s role in tumor suppression. For instance, pancreatic cancer cells that have lowered obscurin expression exhibit increased metastasis (36). Additional studies confirmed that obscurin is expressed at lower levels in a wide variety of tissues at lower levels, and that obscurin knockdown, especially in epithelial cells, increases cellular motility and velocity (35, 37, 38, 39). Mechanistically, this phenotypic change is governed by both the obscurin RhoGEF and PH domains. In epithelia, the obscurin RhoGEF domain activates RhoA, thereby activating the ROCK signaling cascade and leading to decreased cellular migration (9, 36). In another pathway, the obscurin PH domain inhibits the bioavailability and formation of PIP_3_ by both sequestering PIP_2_ in the plasma membrane and by binding to and inhibiting the p85 regulatory domain of PI3K (9, 40, 41). This in turn leads to disruption of the actin cytoskeleton and slower cell velocity.

While obscurin’s mechanism of action has become clearer, several major questions about how obscurin functions in non-muscle cells remain. For instance, obscurin’s subcellular localization in epithelia remains poorly studied, and this impedes a full mechanistic understanding of its function. It is also unclear whether the obscurin C-terminus only interacts with RhoA, PI3K/PIP_3_, and ankyrins, or if there are other physiologically relevant obscurin targets in epithelial cells. Last, and relatedly, the link between obscurin, its immediate downstream effectors, and the modulation of cell migration remains only partially described. To begin filling in these knowledge gaps, we have developed an adenoviral system that delivers a vector containing a tagged version of the C-terminal ∼1000 residues of obscurin into epithelial cell lines. This system shows, for the first time, that the obscurin C-terminus localizes to cell adhesion machinery at the cell membrane. Using various obscurin truncations, we show that the RhoGEF domain, the PH domain, and two regions in the unstructured C-terminal tail are responsible for colocalization to the cell membrane and cell adhesion proteins. We also identify another region of obscurin, at the extreme C-terminus, that modulates PIP_3_ levels. The presence of obscurin at the cell membrane leads to a global actin reorganization, characterized by a decrease in internal stress fibers. Both membrane localization and intact obscurin signaling domains are necessary for this cytoskeletal change. We suggest that this change in actin dynamics directly leads to the cessation of cell motility. Together, these data provide a more complete picture of obscurin’s molecular mechanism of action.

## Results

Obscurin localization and binding partners are most thoroughly characterized in skeletal muscle. In myocytes, obscurin interfaces with ankyrins, RhoA, calmodulin, and PI3K at its C-terminus and titin, myomesin, and MyBP-C at its N-terminus, providing a link between the contractile apparatus and the surrounding membranes (27, 28, 29, 30, 40, 42, 43). Obscurin’s presence in epithelial cells, which lack titin, myomesin, and MyBP-C, raises the question of what it binds to. To begin answering this, we employed an adenoviral delivery system where either the N- or the C-terminus of obscurin A (termed ObscN and ObscA, respectively) with a cassette encoding biotin ligase (BirA) inserted near the middle of the sequence (denoted by Asterix in Fig. 1*A*) was infected into MDCK cells (canine kidney epithelial cells). Besides the expected RhoA, PI3K, calmodulin, and ankyrin, a proximity BioID assay revealed ObscA also interacts with multiple cell adhesion proteins (Fig. 1*B* and Data S1). Interestingly, the protein that mouse ObscA most closely associated with is dog obscurin (79% identical), potentially indicating self-association. Supporting this, native gels of MDCK cells infected with ObscA also show an apparent dimer (Fig.S1). In contrast, ObscN had no strong interaction partners, and does not form dimers in native gels (Data S2 and Fig.S6).

**Figure 2:**
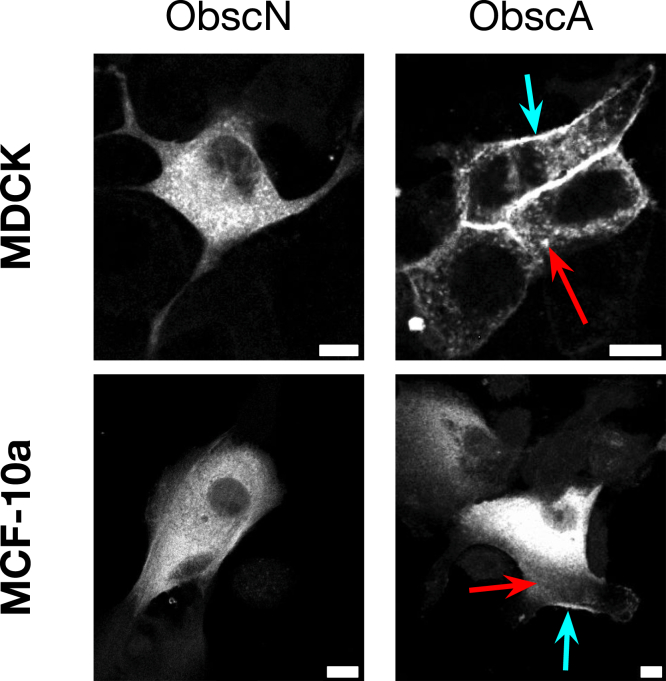
**Obscurin A c-terminus partially localizes to the membrane.** ObscN-mTFP and ObscA-mTFP in MDCK and MCF - 10A cells. *Blue arrows* show cell membrane localization; *red arrows* show punctate cytosolic staining possibly localizing to focal adhesions. Scale bar is 10 μm.

While the ObscA targets RhoA, PIP_3_, and PI3K are at the cell membrane, the enrichment of so many cell adhesion proteins with ObscA was surprising; there is nothing in the literature that suggests mammalian obscurin A colocalizes with cell adhesion junctions (35, 40, 42). However, studies in *Caenorhabditis elegans* suggest an evolutionary precedent for such interactions. The nematode obscurin homolog UNC - 89 associates with integrin-based adhesion complexes through interactions with LIM - 9 (the FHL homolog) and CPNA - 1, which links UNC - 89 to focal adhesion proteins such as UNC - 97 (PINCH) and PAT - 6 (α-parvin) (44, 45). Thus in nematode muscle, UNC - 89 is at integrin adhesion sites where it serves to link adhesion complexes to various sarcomeric components. To verify our results in mammalian epithelial cells, we created new ObscA and ObscN constructs with a gene encoding a dual mVenus-mTFP fluorophore in the biotin ligase location. Confocal images of MDCK and MCF - 10A cells infected with ObscN reveal a diffuse cytosolic localization pattern with occasional puncta (Fig. 2). In contrast, while cells containing ObscA also show some cortical and cytosolic localization, most cells also exhibit clear cell membrane localization. This ability of ObscA to localize to the membrane, and possibly to focal adhesions, was further confirmed by infecting ObscA into focal adhesion-deficient ZO - 1 KO MDCK cells (Fig. S7) (46). In these cells, ObscA is not membrane-associated.

**Figure 3:**
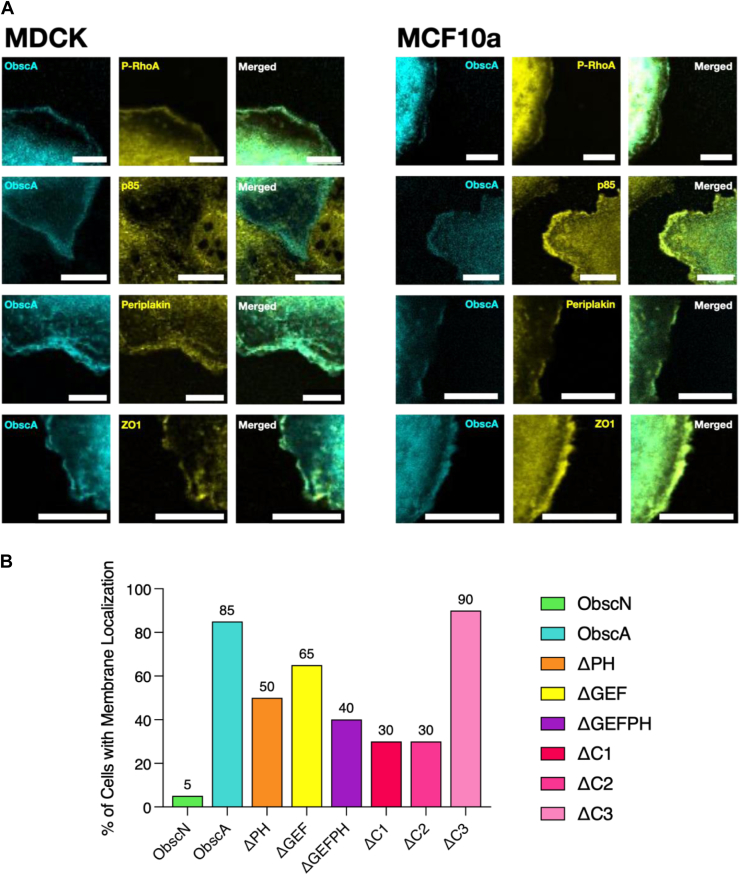
**Obscurin localizes to regions of cell adhesion in the membrane.***A*, ObscA-mTFP-infected MDCK and MCF - 10A cells stained with various antibodies to detect colocalization. Obscurin colocalizes with the desmosome (periplakin), tight junctions (ZO1), PI3K (p85), and RhoA at or near the membrane. *B*, the percentage of cells infected with ObscN (n = 79), ObscA (n = 63), ΔPH (n = 43), ΔGEF (n = 46), ΔGEFPH (n = 53), ΔC1 (n = 57), ΔC2 (n = 45), and ΔC3 (n = 45) constructs that display obscurin localization at or near the cell membrane in MDCK cells. ObscN, ΔGEFPH, ΔC1, and ΔC2 were mainly cytosolic, while ObscA, ΔPH, ΔGEF, and ΔC3 were primarily located at or near the membrane.

To verify the ObscA cell membrane localization and to further explore the BioID results, we next performed a series of colocalization microscopy studies. Examples of membrane localization binning can be found in Fig. S8. As expected, ObscA colocalizes with phosphorylated (active) RhoA at or near the membrane and, to a much lesser extent, with the p85 domain of PI3K (thresholded Mander’s coefficients of 0.94 and 0.44, respectively) (Figs. 3*A* and S5). Additionally, ObscA strongly colocalizes with proteins at the desmosome (periplakin) and tight junctions (ZO1) (thresholded Mander’s coefficients of 0.98 and 1.00). Similar colocalization patterns were observed in both MDCK and the human-derived breast epithelial cell line MCF - 10A, suggesting that these patterns are widely applicable to epithelial cells across species.

We next asked which regions of obscurin are responsible for this cell membrane colocalization. We created a series of fluorescent ObscA deletion mutants (Fig. 1*A*) and infected these into both MDCK and MCF - 10A cells. In cells containing ObscA, fluorescence was present at the membrane about 85% of the time. In the remaining 15%, the fluorescence was more cortical, and generally diffuse within the cytosol (Fig. 3*B*). In contrast, removal of the PH domain, which abolishes interactions with both PIP_3_ and PI3K, dropped the percentage of cells with membrane-associated fluorescence to around 50%. However, in the instances where this ΔPH construct was observed at the membrane, it exhibited the same colocalization partners as the full length ObscA construct (Fig. S9*A*). Likewise, the construct with the removal of the RhoGEF domain (ΔGEF) is present at the cell membrane in only 65% of the cells imaged. This is likely explained by the tendency of the RhoGEF binding partner, RhoA, to colocalize at or near the leading edge of migrating cells (9, 10). Once again, in instances when the ΔGEF construct was observed at the membrane, it colocalizes with the same partners as the full length ObscA construct, including with activated RhoA (Fig. S9*B*). Western blot analysis using anti-mVenus did not show any obvious protein degradation in any of the ObscA constructs.

Deletion of both the RhoGEF and the PH domain simultaneously (ΔGEFPH) did not fully ablate ObscA membrane localization (Figs. 3*B* and S9*C*). The remaining regions of ObscA consist of two Ig domains with no known binding partners and a ∼350 residue intrinsically unstructured domain. This unstructured region contains an ankyrin binding site, and we reasoned that this segment may partially contribute to the residual membrane localization of the ΔGEFPH construct. In colocalization experiments where the ankyrin site was deleted from the otherwise-full length ObscA construct (residues 7261–7419, corresponding to residues 6383–6532 in human obscurin A; termed ΔC2), 30% of cells exhibited fluorescence at the membrane (Figs. 3*B* and S9*E*). Deletion of an area more N-terminal to the ankyrin binding site (7077–7260, corresponding to 6199–6382 in human obscurin A; termed ΔC1) also resulted in a case where 30% of the cells displayed obscurin membrane localization. In contrast, around 90% of the cells exhibited fluorescent cell membrane localization when the C-terminal 76 residues were removed (ΔC3) (Fig. 3*B*). In each deletion construct, those cells that had fluorescent membrane localization had the same colocalization partners as with the original ObscA construct (Fig. S9, *D*–*F*). While the lack of membrane localization in ΔC2 can be explained by the ablation of the ankyrin binding sequence, the lack of membrane localization with ΔC1 was unexpected, and may represent a new motif that partially directs obscurin to specific membrane regions involved in cell adhesion. Overall, these data suggest obscurin has multiple regions responsible for membrane localization, and that the avidity from these combined elements to membrane targets, rather than the affinity of one element, drives cell membrane localization.

**Figure 4:**
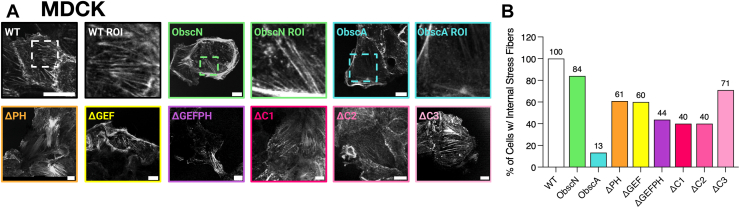
**Obscurin decreases the number of stress fibers in MDCK cells.***A*, representative images for each treatment in MDCK cells; scale bar is 10 μm. *B*, total approximate percentage of central stress fibers present in WT (n = 89), ObscN (n = 57), ObscA (n = 69), ΔPH (n = 48), ΔGEF (n = 55), ΔGEFPH (n = 60), ΔC1 (n = 79), ΔC2 (n = 81), and ΔC3 (n = 56) in MDCK cells. All cells chosen for this analysis also had obscurin at the periphery of the cells.

A recent paper by Eason *et al.* found that a myristilated version of an obscurin-PH construct that localizes to the cell membrane, but not a non-myristilated version that localizes to the cytosol, disrupts the actin cytoskeleton (41). Having previously established that four obscurin regions (RhoGEF, PH, C1, and C2) are partially responsible for membrane localization, we next explored which regions also alter cellular actin. The binning process for actin analysis can be found in Fig. S10. Phalloidin staining showed that almost all MDCK and MCF - 10A cells possess an extensive internal stress fiber network (Figs. 4*A*, S11, and S12). There are some differences between these cell types; MDCK cells tend to have higher percentages of central actin fibers than MCF - 10A cells, and MCF - 10A cells have a more extensive filopodia network. In both cell types, infection with the ObscN construct does not noticeably change the actin staining morphology, confirming that our adenoviral infection system does not significantly alter this aspect of the actin cytoskeleton (Figs. 4*B*, S11, and S12). In contrast, only about 13% of all cells infected with ObscA have central stress fibers. Instead, actin staining is more peripheral. This phenotype has been reported previously in RhoA-inhibited fibroblasts (47).

We next explored which obscurin regions are responsible for this change in the actin cytoskeleton morphology. We first studied the stress fiber staining pattern of ΔC1 and ΔC2- infected cells and found that cells without ΔC1 and ΔC2 present at the membrane tend to also contain centralized stress fibers. We deduced that the ObscA’s failure to induce actin reorganization is due to incorrect ObscA localization. Extrapolating from this, we limited ourselves to only studying the actin morphology only in cells where the various ObscA constructs were membrane-localized (Figs. 4, S11, and S12).

Infection of either ΔPH or ΔGEF results in a population of cells that are midway between the WT and the ObscA actin phenotype. This suggests that these two obscurin regions are involved in but are not solely responsible for stress fiber regulation (Figs. 4 and S11, and S12). Our colocalization and BioID data show that obscurin does not bind to actin directly. Instead, previous studies have shown that both the PH and the RhoGEF initiate cytoskeletal rearrangement pathways (9, 42). Based on this evidence, we reasoned that infection with the ΔGEFPH construct should result in cells with an actin cytoskeleton morphology similar to that seen in ObscN-infected cell cultures. However, cell cultures infected with ΔGEFPH had many fewer cells with central stress fibers than ObscN-infected cells (*p* < 0.05). We interpreted this to indicate the existence of a third obscurin region that alters cellular actin architecture. Accordingly, infection with the ΔC3 construct resulted in cells with internal actin fibers, similar to ObscN-infected cells (*p* > 0.1). These data strongly suggest this region influences actin cytoskeletal dynamics (Figs. 4 and S12, *A* and *B*).

**Figure 5:**
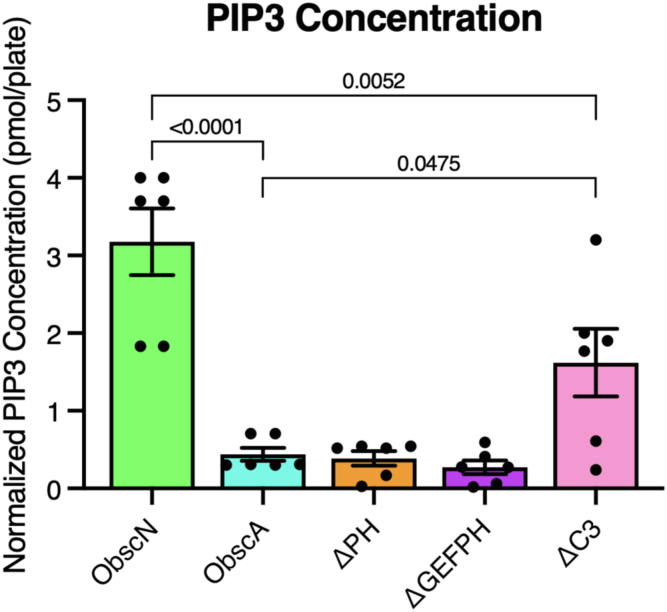
**Obscurin domains variably affect whole-cell PIP_3_ levels.** PIP_3_ amounts in MDCK cells (n = 6 for all conditions).

To further examine why infection with ΔC3 alters stress fiber formation, we quantified the amount of PIP_3_ present in a series of obscurin-truncation infected MDCK and MCF - 10A plates. In motile cells, PIP_3_ activates the mTORC1 and mTORC2 pathways, leading to increased actin polymerization and faster cell migration (9, 17, 48). Previous research has shown that the obscurin PH domain decreases PIP_3_ levels through both a direct sequestration mechanism and through binding and subsequent inhibition of the p85 regulatory domain of PI3K. We wondered if the extreme C-terminus feeds into this same pathway. As expected, cells infected with either ObscA or ΔGEF had lower PIP_3_ levels, as compared to ObscN-infected cells (Figs. 5 and S13). However, infection with ΔPH or ΔGEFPH did not rescue PIP_3_ levels, suggesting the presence of an additional PIP_3_ modulator in the obscurin C-terminus. Infection with the ΔC3 construct resulted in increased PIP_3_ levels. These data suggest that along with the previously-described PH domain, the C3 region of obscurin also dampens PIP_3_ levels through an unknown binding partner, and the C3 region modulates actin stress fiber morphology through this intermediary.

**Figure 6:**
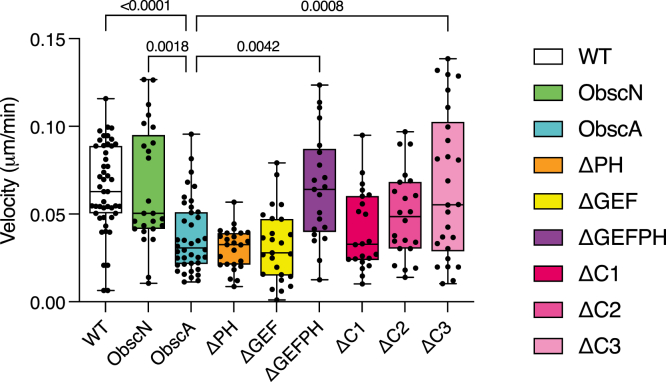
**Obscurin constructs variably influence MDCK cell motility.** WT (n = 60), ObscN (n = 30), ObscA (n = 40), ΔPH (n = 25), ΔGEF (n = 25), ΔGEFPH (n = 23), ΔC1 (n = 23), ΔC2 (n = 25), and ΔC3 (n = 28). *p*-values are displayed at the top.

Multiple studies have shown that treatment with obscurin siRNA drastically increases cultured epithelial cell motility and converts cells to a more mesenchymal phenotype (38, 39, 49, 50). To test whether overexpression of the C-terminus of obscurin has the opposite effect, we infected both MDCK and MCF - 10A cells with the ObscA or ObscN construct and measured cell motility. Infection with ObscN did not change the rate of cell movement, indicating that the adenovirus infection itself does not perturb motility (Fig. 6). Infection with ObscA resulted in significantly decreased cell migration. This effect was more pronounced with MDCK cells, likely due to MCF - 10A cells being less motile at baseline in our experimental conditions (data not shown).

Given that the obscurin RhoGEF, PH, and C3 regions are associated with an altered actin cytoskeleton, we hypothesized that infection of obscurin constructs containing a deletion of each of these regions would at least partially restore cell motility. This is in fact the case with both ΔC3 and ΔRhoGEF-infected cells (Fig. 6). Infection with either ΔGEF or ΔPH alone did not increase motility. This pattern suggests that even though the RhoGEF and PH domains activate separate pathways, the downstream effect on cell motility is redundant and cell migration is only rescued when both domains are removed.

## Discussion

Here we infected epithelial cells with constructs encoding the fluorescently-labeled or BirA-labeled obscurin A C-terminus. This method presents multiple advantages over existing procedures to study this protein. Anti-obscurin antibodies tend to demonstrate poor affinity in immunohistochemical preparations, and the presence of multiple obscurin isoforms has hindered a full understanding of obscurin’s location in epithelial cells (25, 35, 51). These issues are not present in the adenoviral system described in this paper, where obscurin composition is easily controlled, can be easily checked, and is easily visualized. This method also allows for live-cell imaging of obscurin localization under various conditions, and provides an orthogonal technique to yeast-2-hybrid experiments for determining obscurin binding partners (22, 27). In addition, introducing exogenous obscurin into a live system complements other studies where endogenous obscurin is knocked down *via* siRNA methods (25, 38, 39). The main drawbacks of this strategy are that it utilizes obscurin overexpression, and only uses part of the full-length obscurin molecule. Obscurin overexpression causes obscurin to accumulate cortically and/or nuclearly. This produces experimental caveats, such as the BioID proximity assay identifying several obscurin-proximal proteins that are unlikely to be true binding partners, including transcription factors, ribosomal proteins, and nuclear pore proteins (Datas S1 and S2) (35).

**Figure 7:**
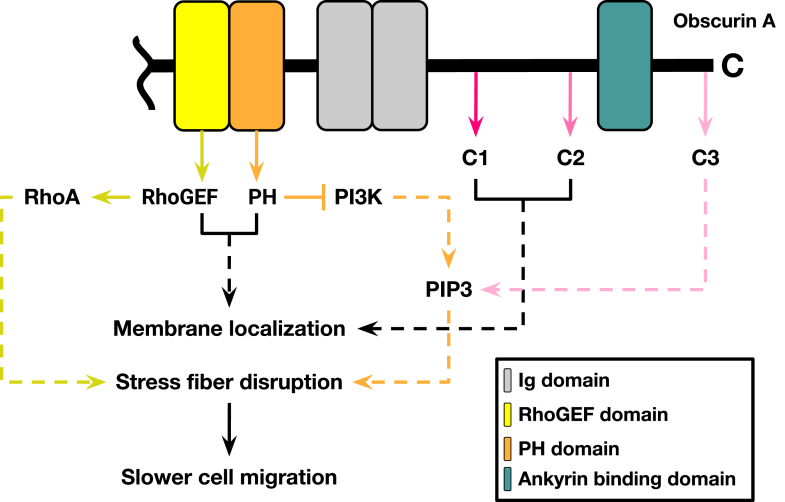
**Mechanisms involved in obscurin’s localization and stress fiber disruption.** The RhoGEF, PH, C1, and C2 regions influence membrane localization while the RhoGEF, PH, and C3 regions influence stress fiber formation. The PH and C3 region do this by influencing PIP_3_ levels, while the RhoGEF region was previously shown to influence RhoA activation. Together, these three parallel pathways reduce cell migration. *Dashed lines* represent facets of this mechanism explored in this study while solid lines are data from previous studies.

Given the obscurin A binding partners that we and others have identified, it was not surprising that the ObscA constructs described here localized at or near the cell membrane. Our data suggest this localization is driven by four regions: the RhoGEF domain, the PH domain, the C2 region, and the C1 region (Figs. 1*B* and 7). The RhoGEF domain binds to RhoA, which is membrane-adjacent in its active form (52). The obscurin PH domain both binds to PI3K, which localizes at the cell membrane, and also binds to the phospholipid PIP_3_ (40, 50). The C2 region has been previously shown to interact with ankyrin G, which in turn associates with desmosomes, adherens junctions, and gap junctions (27, 53, 54, 55). In contrast, the C1 region is not known to associate with any targets, yet the ΔC1 construct exhibits significant loss of membrane localization, suggesting that there are other as of yet undefined interactions that are partially responsible for obscurin’s localization pattern in epithelial cells. These data, combined with the BioID proximity assay, indicate a much richer obscurin interactome than previously realized.

The addition of the ObscA construct causes an alteration in stress fiber morphology in both MDCK and MCF - 10A cells. Actin neither colocalizes with obscurin nor was it identified as an obscurin target in the BioID proximity assay, suggesting that obscurin’s effect on stress fibers is indirect. Obscurin likely affects the cytoskeleton through previously defined RhoA and PIP_3_ pathways (9). Both of these effectors influence actin polymerization and, indirectly, cell velocity in a temporal and spatial-dependent manner (9, 10). Given this intricate signaling web governing cell motility, it is perhaps not surprising that obscurin-driven alterations to actin growth and actomyosin contraction dampens cell velocity.

The obscurin C-terminus contains at least three regions that ultimately serve the redundant purpose of decreasing cell velocity-the RhoGEF domain, the PH domain, and the extreme C terminus (C3). Given obscurin’s nexus for cell motility signaling, this could explain why it is so often mutated in cancers and highlights its therapeutic potential. Since each signaling domain can suppress motility (Fig. 7), each obscurin segment can be independently harnessed to alter cell velocity. For instance, the addition of the obscurin PH domain prevents epithelial metastasis through a variety of downstream effectors (41). Similarly, the loss of the obscurin RhoGEF domain downregulates RhoA activity, leading to increased micro-tentacle formation and increased metastasis in multiple cell types (36, 38, 49). It is likely that a stand-alone C3 region would be similarly effective, and this will be the subject of future experimentation. A construct with all three of these anti-migration factors, along with a membrane localization signal, is an attractive potential scaffold for a multipronged antimetastatic therapeutic.

## Experimental procedures

### Cell lines and culturing

Madin-Darby Canine Kidney (MDCK) and MCF10 A non-tumorigenic, immortal epithelial cell lines were used in all experiments (Fig. S1). MDCK cells were cultured using Dulbecco’s Modified Eagle’s Medium (DMEM) supplemented with 5% Fetal Bovine Serum (FBS) and Penicillin/Streptomycin (P/S) for optimal growth conditions. MCF10 A cells were cultured using MCF10 A Cells Complete Medium (MSE Supplies, Tucson AZ). Cells were plated on 35 mm^2^ glass bottomed dishes (Mattek, Ashland MA) and incubated at 37 °C with 5% CO_2_ until use.

### Virus generation and constructs

All obscurin constructs were constructed from mouse obscurin A; protein accession number NP_954603. Codon optimized DNA encoding either a biotin ligase construct or a mTFP-mVenus construct (56) was inserted in-frame before the DNA encoding Ig5 for ObscN, or directly after the DNA encoding Ig67 for all other constructs. Obscurin fragments encoded by these constructs ran ∼115 kDa on 12% SDS-gels, as confirmed by an a-Venus Western blot (data not shown). DNA constructs were incorporated into an adenoviral vector under a CMV promoter by Vectorbuilder (Chicago IL).

### Infection

Directly after cell trypsinization and reconstitution, adenovirus-containing obscurin constructs were added to cell culture dishes MDCK or MCF - 10A cells at a 1:10 MOI. Cells were incubated for 24 to 48 h and monitored using mTFP1 fluorescence (492 nm) to verify that there was a minimum of 60% infection-rate.

### BioID Assay

MDCK cells were infected on a 100 mm^2^ dish with either ObscA, ObscN, or a sham control and dosed with biotin. At 48 h, when cells were 70 to 100% confluent, cells were washed, lysed with RIPA buffer + AEBSF. The resulting supernatant was incubated with streptavidin beads and washed 3x in RIPA buffer. 10% of the bead slurry was run on an SDS-PAGE gel to verify a unique banding pattern, and the remaining beads were sent for MS/MS analysis (MS BioWorks, Ann Arbor MI).

### Colocalization

MDCK and MCF - 10A cells at 40 to 60% confluency or higher were fixed using 4% paraformaldehyde, permeabilized with 0.1% Triton-X 100, and blocked with 2% BSA in PBS. A 1:100 dilution of primary antibody and 1:500 dilution of the appropriate AlexaFluor - 647-conjugated secondary antibody was added (Fig. S2). Plates were mounted with Prolong Gold, covered with 0.22 mm glass cover slips, and imaged using the Leica Stellaris eight Laser-Scanning confocal microscope. Between 15 to 30 images of cells over 2 to 5 experimental plates were taken per treatment group.

### Actin staining

Cells were prepared as described, with the addition of 1:500 Phalloidin iFluor - 594 added after fixing. A minimum of 30 cells were imaged on the Leica Stellaris eight laser-scanning confocal microscope for data analysis. All images were exported for analysis in ImageJ. Additional information about figure-specific microscopy methods about information can be found in the supplemental materials and methods (57).

### Velocity

Cells at ∼40% confluency were stained using Orange Cytopainter (Abcam; Cambridge UK) and allowed to sit in a solution of FluoroBrite medium (Thermo; Waltham MA) with Trolox. Plates were imaged using the Nikon Eclipse Ti2 widefield microscope and analyzed in ImageJ using the Trainable WEKA Segmentation and Mtrack2 plugins as previously described (58). A power analysis indicated a required minimum of 23 cells per treatment (Fig. S3). Statistical significance was determined using a *p*-value = 0.05.

### Microscopy

Colocalization experiments were collected on a Leica Stellaris eight Laser-Scanning confocal microscope running Leica Application System X (LASX) software with a HC PL APO CS2 63x/1.40NA oil-immersion objective. Obscurin was visualized using the mTFP1 (Ex/Em = 462/492) channel with a laser line at 462 nm and the detection range was 487-571 nm, secondary antibodies were imaged using the AlexaFluor - 647 (Ex/Em = 650/665) channel with a laser line at 633 nm and the detection range was 660-720 nm. 8 bit single images of both channels were generated simultaneously, with 1 Airy Unit pinhole size. A sub - 10% laser intensity was maintained throughout imaging. Velocity data were collected using a Nikon Eclipse Ti - 2 widefield microscope with a Plan Fluor 40x/1.30NA oil-immersion objective and running Nikon Elements software. Imaging was done using the Hamamatsu ORCA Flash 4.0, and all images are 12 bit. Fluorescence of orange cytopainter (Ex/Em = 590/700) and mTFP1 (Ex/Em = 462/520) was used for the velocity data. Five areas per sample per timelapse were selected for imaging every 10 or 5 min (MDCK cells and MCF - 10A cells, respectively) for a period of 10 or 5 h, respectively. Plates for velocity data were housed in a Tokai-Hit Incubation Chamber during imaging, set to 37 °C with 5% CO_2_ circulation. Additional information about figure-specific microscopy methods can be found in the supplemental materials and methods and Fig. S4.

### Image analysis

To quantify colocalization between constructs and antibodies, the Colocalization Threshold plugin from ImageJ was utilized using Costes’ auto-thresholding and Manders’ coefficient to describe colocalization patterns. The ROI, chosen to be near the membrane, was the only area analyzed from the images and brightness/contrast were not changed. The data output included tM1 and tM2 values, which are auto-thresholded to reduce the effect of background noise and shadows. Colocalization was also analyzed using a thresholded Manders’ correlation coefficient (59). Actin fiber analysis binning process is described in Fig. S5. Prism GraphPad was used to make bar graphs and box plots, along with statistical analyses (ordinary one-way ANOVA for multiple comparisons).

### PIP_3_ assay

Cells infected with adenovirus-containing obscurin constructs were grown to 90% confluency in a six-well dish and harvested using the manufacturer’s instructions (PIP_3_ Mass ELISA assay kit; Echelon Biosciences). PIP_3_ standards and samples were run in triplicate to determine total PIP_3_ per well. Duplicate rows of PIP_3_ standards are used to generate a calibration curve. All data were normalized to cell count.

## Data availibility

Most data supporting the findings of this study are included in the manuscript and the supporting information. Additional data, including original microscopy images, are available upon request to Nathan Wright at wrightnt@jmu.edu.

## Supporting information

This article contains supporting information.

## Conflict of interest

The authors declare that they have no conflicts of interest with the contents of this article.
