# Supplementary material for: Obscurin a localizes near the cell membrane to modulate stress fiber dynamics and cell migration
Source: J Biol Chem. 2026 Mar 17;302(5):111383. doi: 10.1016/j.jbc.2026.111383 (PMC13100297; doi:10.1016/j.jbc.2026.111383)
Supplement: Supplementary material [file mmc1.pdf]

Supplemental Figure 1

Cell Line Information		
**Name**	**Supplier**	**Cat #**
MCF10A; Breast; Human	ATCC	CRL10317
MDCK.2; Kidney; Dog (Canis *familiaris*)	ATCC	CRL2936

Supplemental Figure 2

Primary and Secondary Antibodies			
**Name**	**Supplier**	**Cat #**	**Dilution**
B-catenin Monoclonal Antibody (OTI1G1)	Origene	TA502299	1:100
PPHLN1 Polyclonal Antibody	Invitrogen ™	PA544845	1:100
PI3K p85 α-Polyclonal Antibody	Invitrogen ™	PA588089	1:100
Phospho-RhoA (Ser188) Rabbit anti-Human	Bioss	BS5330R	1:100
ZO-1 Polyclonal Antibody	Invitrogen ™	617300	1:100
Goat Anti-Mouse IgG H&L (Alexa Fluor® 647)	Abcam	Ab150115	1:500
Goat Anti-Rabbit IgG H&L (Alexa Fluor® 647)	Abcam	ab150079	1:500

Supplemental Figure 3

```
# Install the pwr package if not already installed
install.packages("pwr")
# Load the pwr package
library(pwr)

calculate_cohens_d <- function(mean1, mean2, sd1, sd2)
{pooled_sd <- sqrt((sd1^2 + sd2^2) / 2)
d <- (mean1 - mean2) / pooled_sd
return(d)}

calculate_power_diff_n <- function(mean1, mean2, sd1, sd2, n1, n2, sig.level = 0.05)
{# Calculate Cohen's d
  d <- calculate_cohens_d(mean1, mean2, sd1, sd2)

  # Perform the power analysis using pwr.2p2n.test for different sample sizes
  power_result <- pwr.2p2n.test(n1 = n1, n2 = n2, h = d, sig.level = sig.level)

  return(power_result$power)}

# Your specific values
mean1 <- 0.0786
mean2 <- 0.037
sd1 <- 0.056
sd2 <- 0.020594155
n1 <- 20  # Sample size for group 1
n2 <- 40  # Sample size for group 2 (example value, adjust as needed)
sig.level <- 0.05

# Calculate the power
power <- calculate_power_diff_n(mean1, mean2, sd1, sd2, n1, n2, sig.level)
print(paste("Power:", power))
```

Supplemental Figure 4

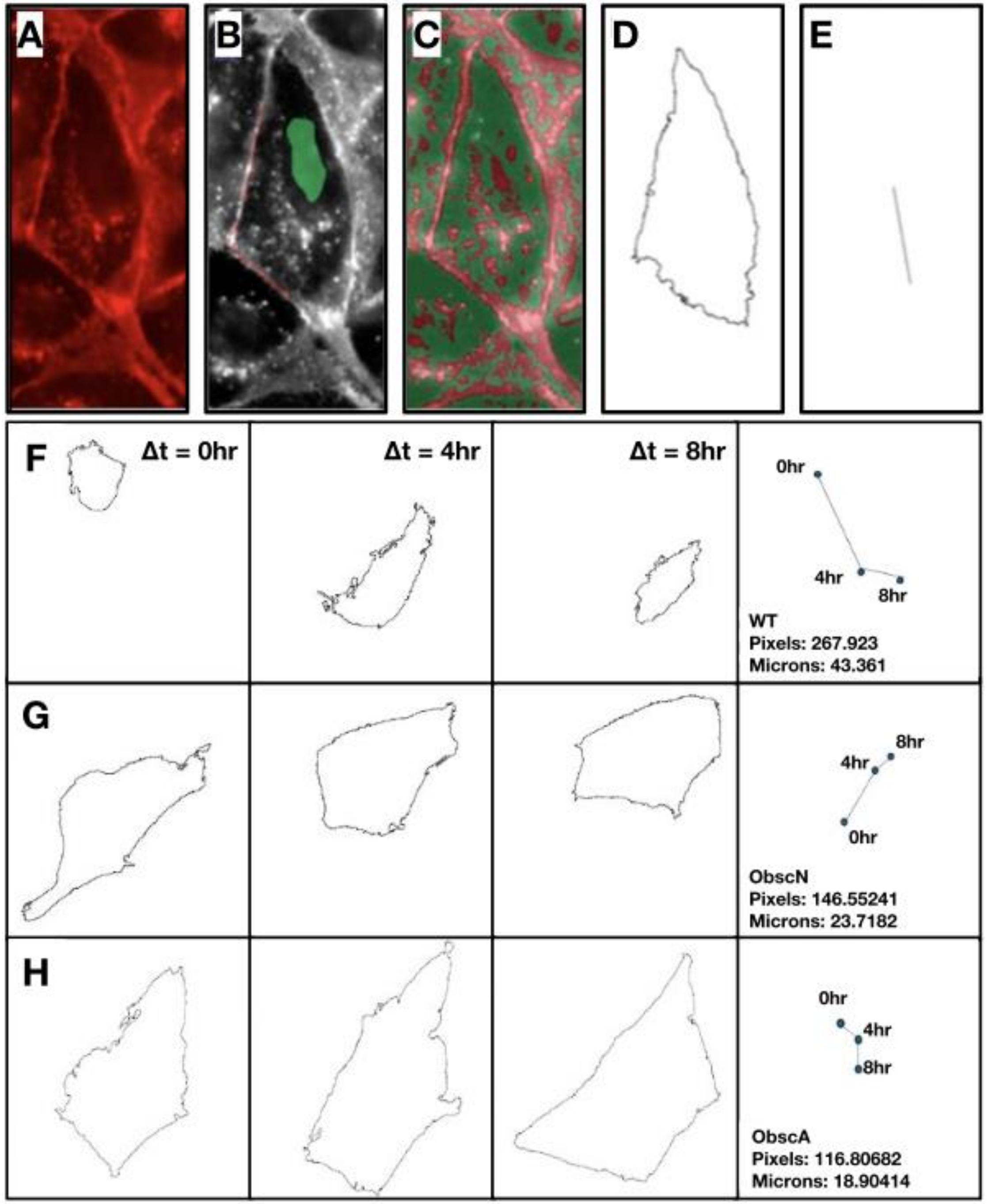

Supplemental Figure 5

Manders' Coefficient Values			
**Treatment**	**MDCK**	**MCF10a**	**Interpretation**
**ObscA + Periplakin**	tM1 = 0.9818<br>tM2 = 0.9201	tM1 = 0.9243<br>tM2 = 0.9026	Strong in both
**ObscA + ZO1**	tM1 = 1.000<br>tM2 = 0.9663	tM1 = 0.9986<br>tM2 = 0.9853	Strong in both
**ObscA + P85**	tM1 = 0.1005<br>tM2 = 0.0296	tM1 = 0.9252<br>tM2 = 0.9245	Weak in MDCK,<br>Strong in MCF10a
**ObscA + P-RhoA**	tM1 = 0.9450<br>tM2 = 0.7246	tM1 = 0.9986<br>tM2 = 0.9853	Strong in both

Supplemental Figure 6

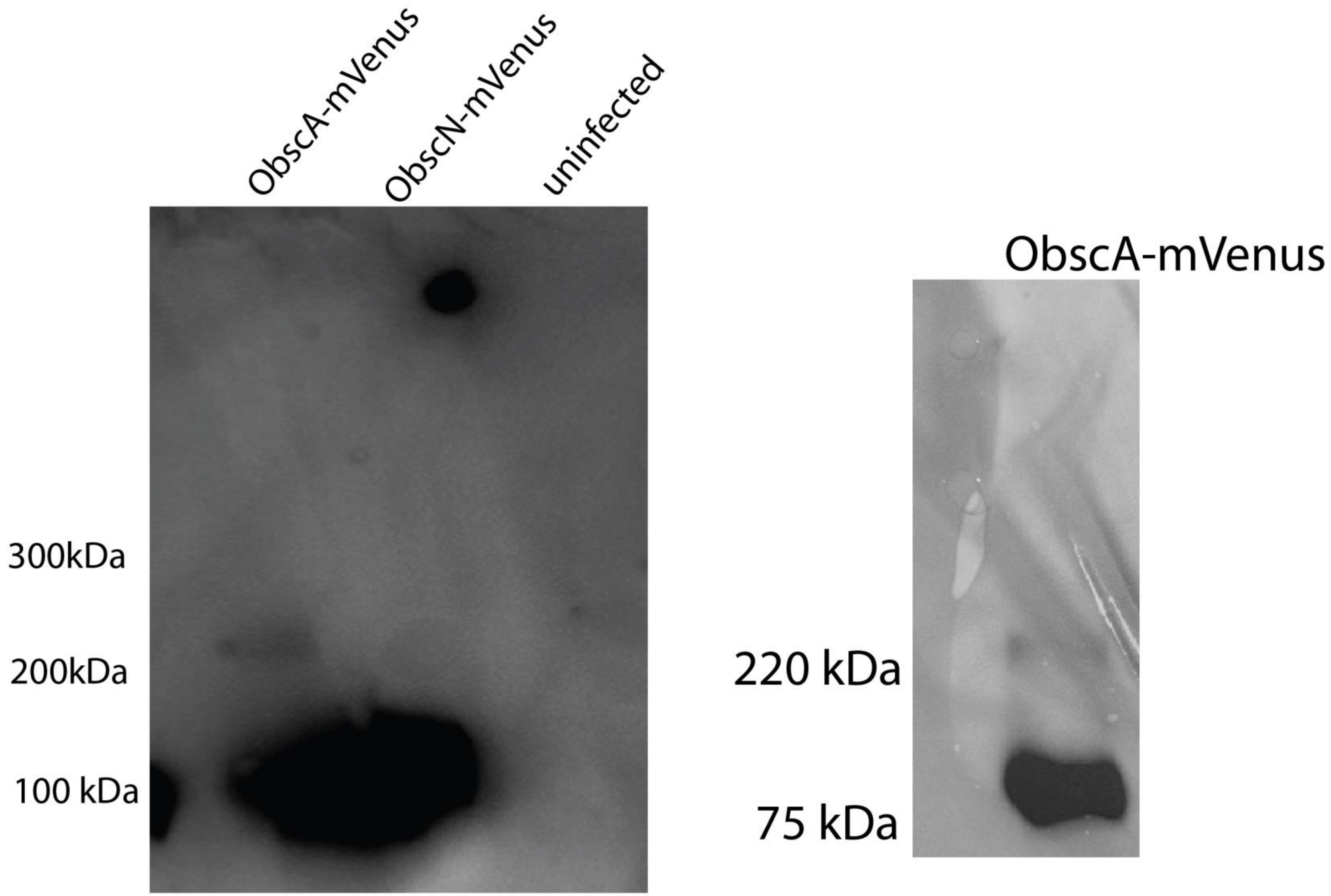

Supplemental Figure 7

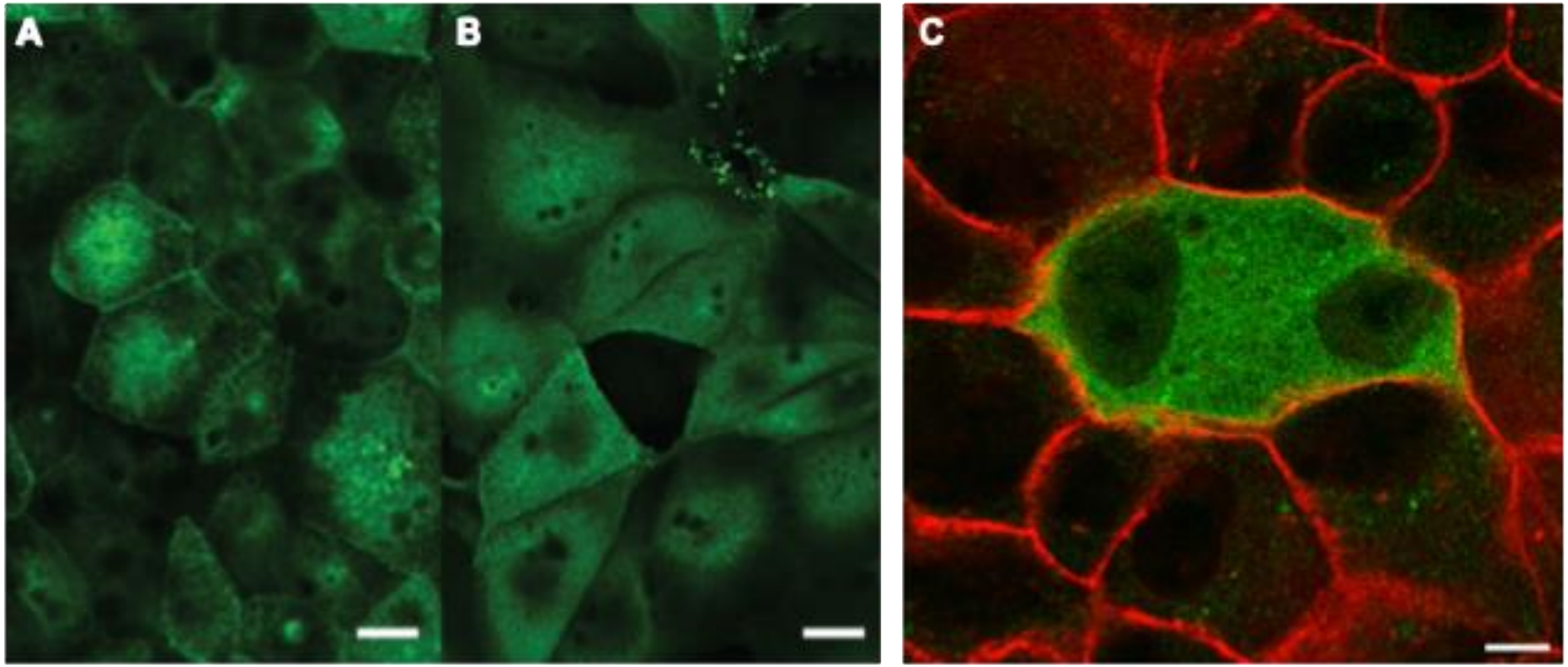

Supplemental Figure 8

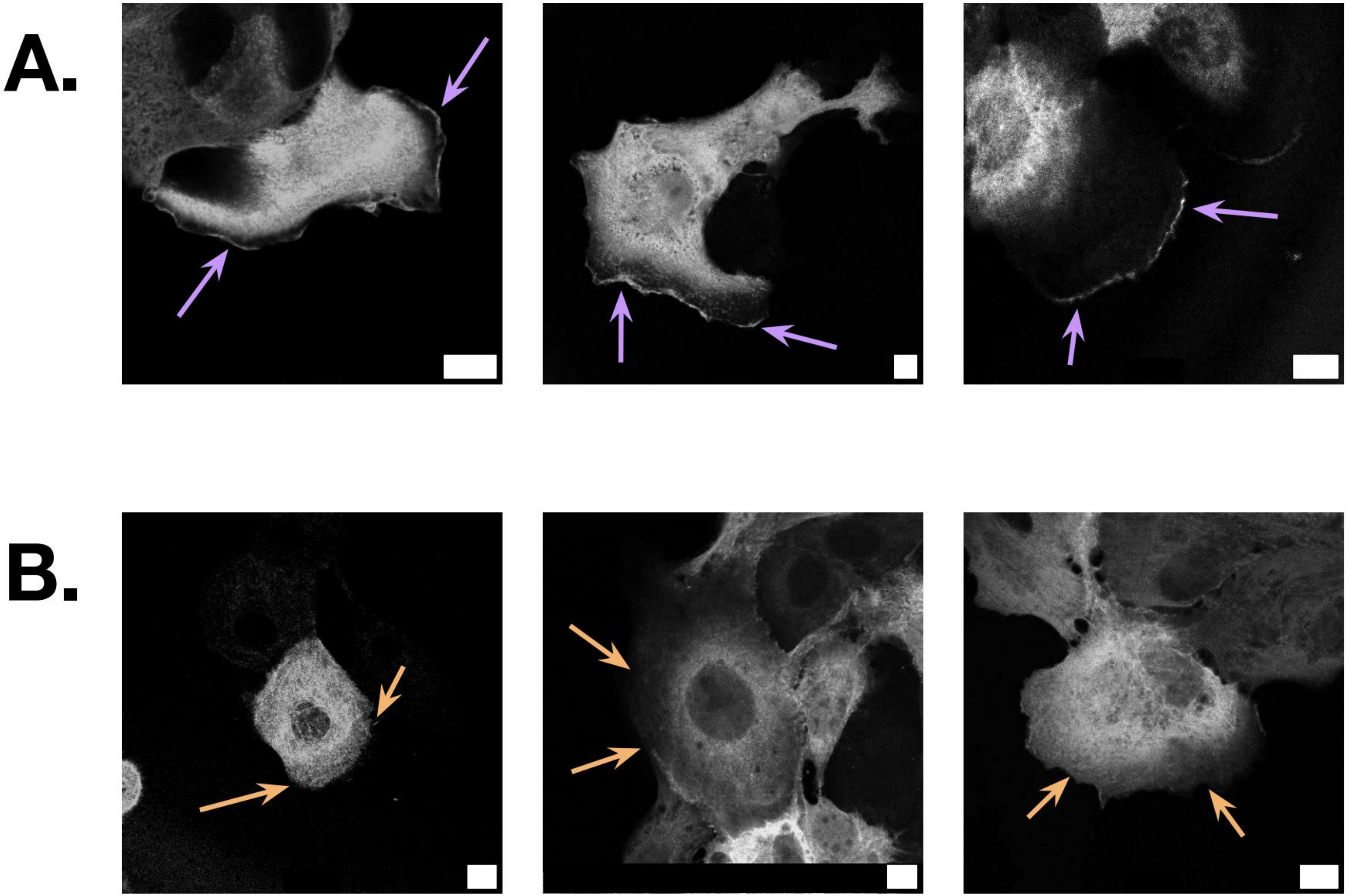

Supplemental Figure 9A

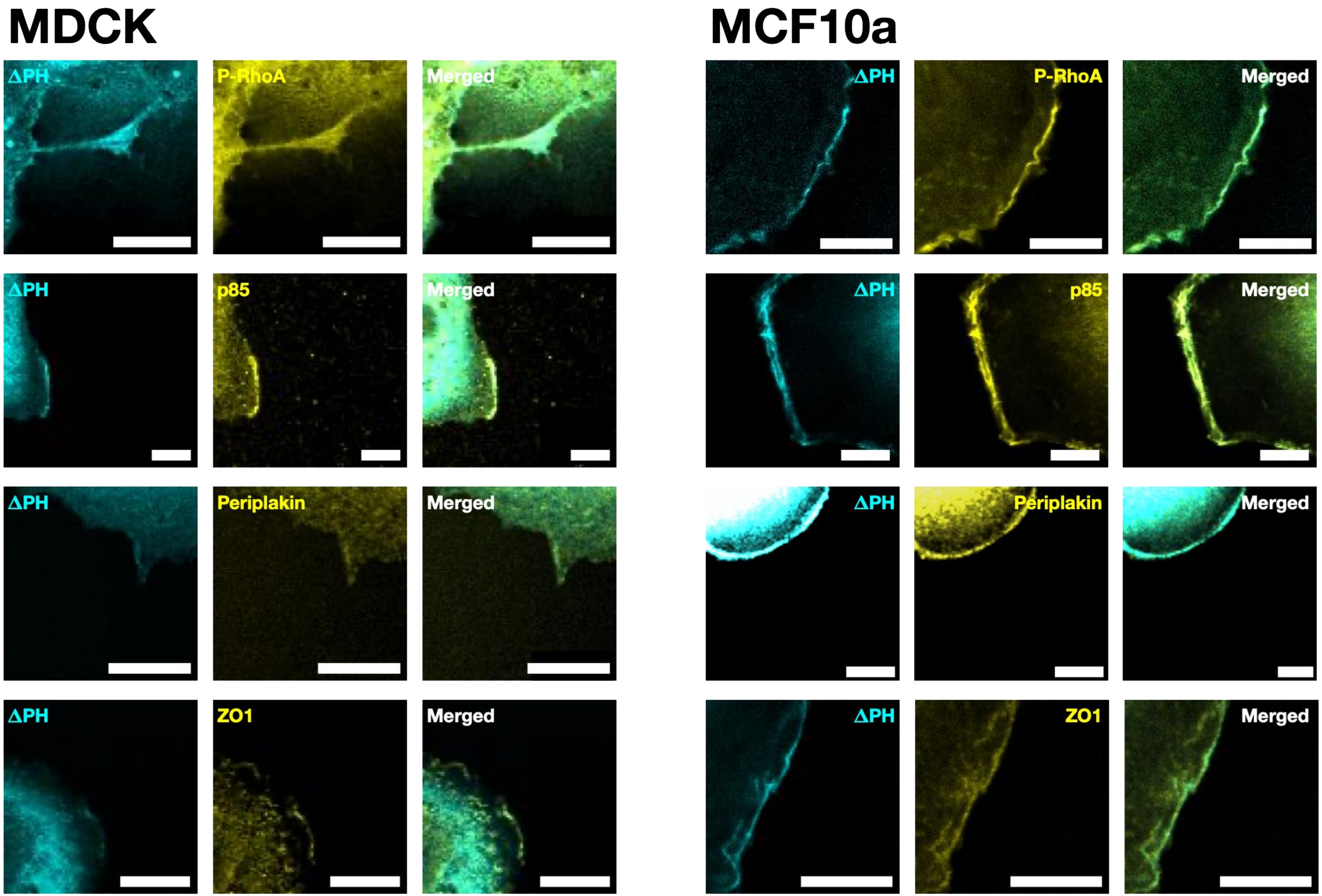

Supplemental Figure 9B

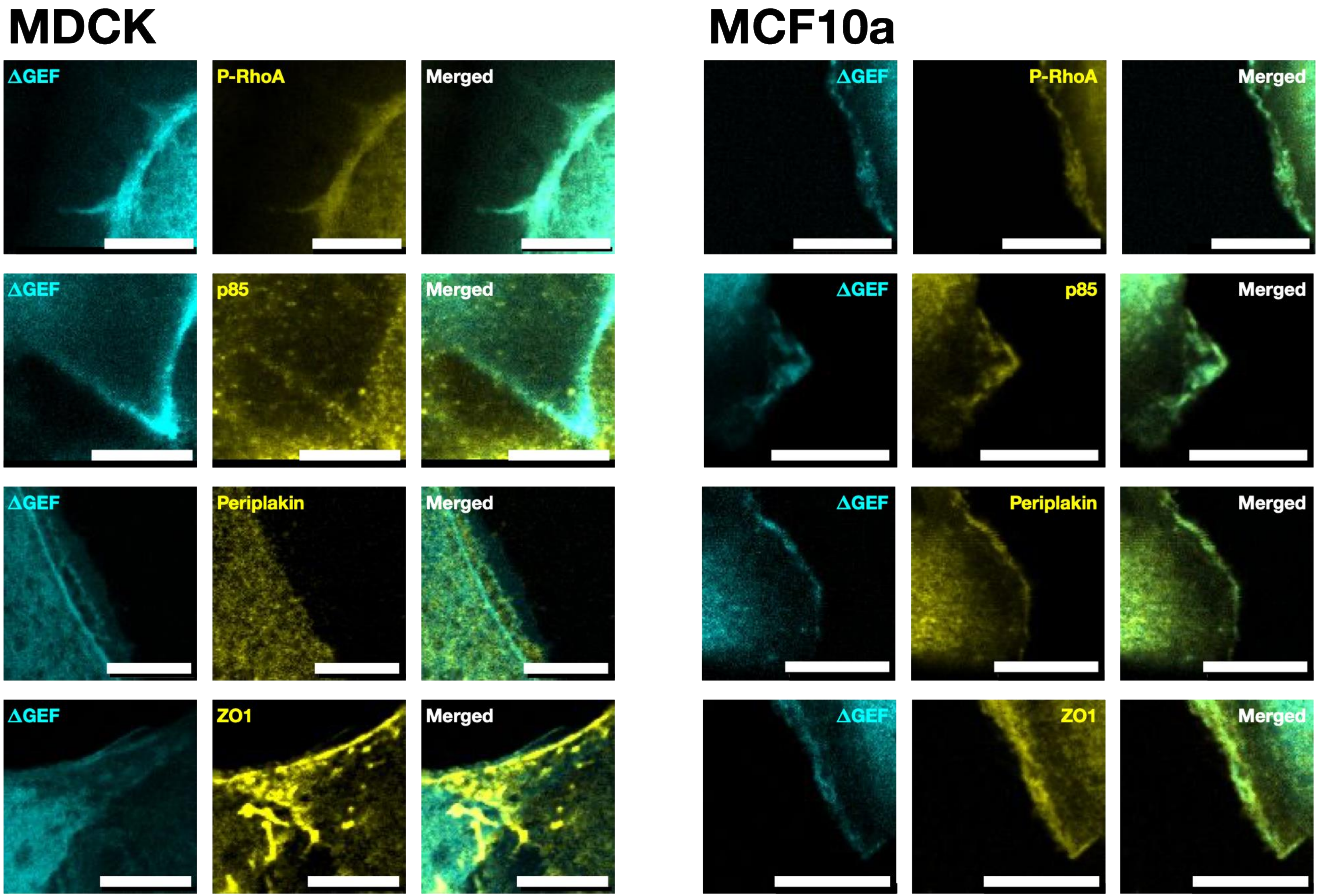

Supplemental Figure 9C

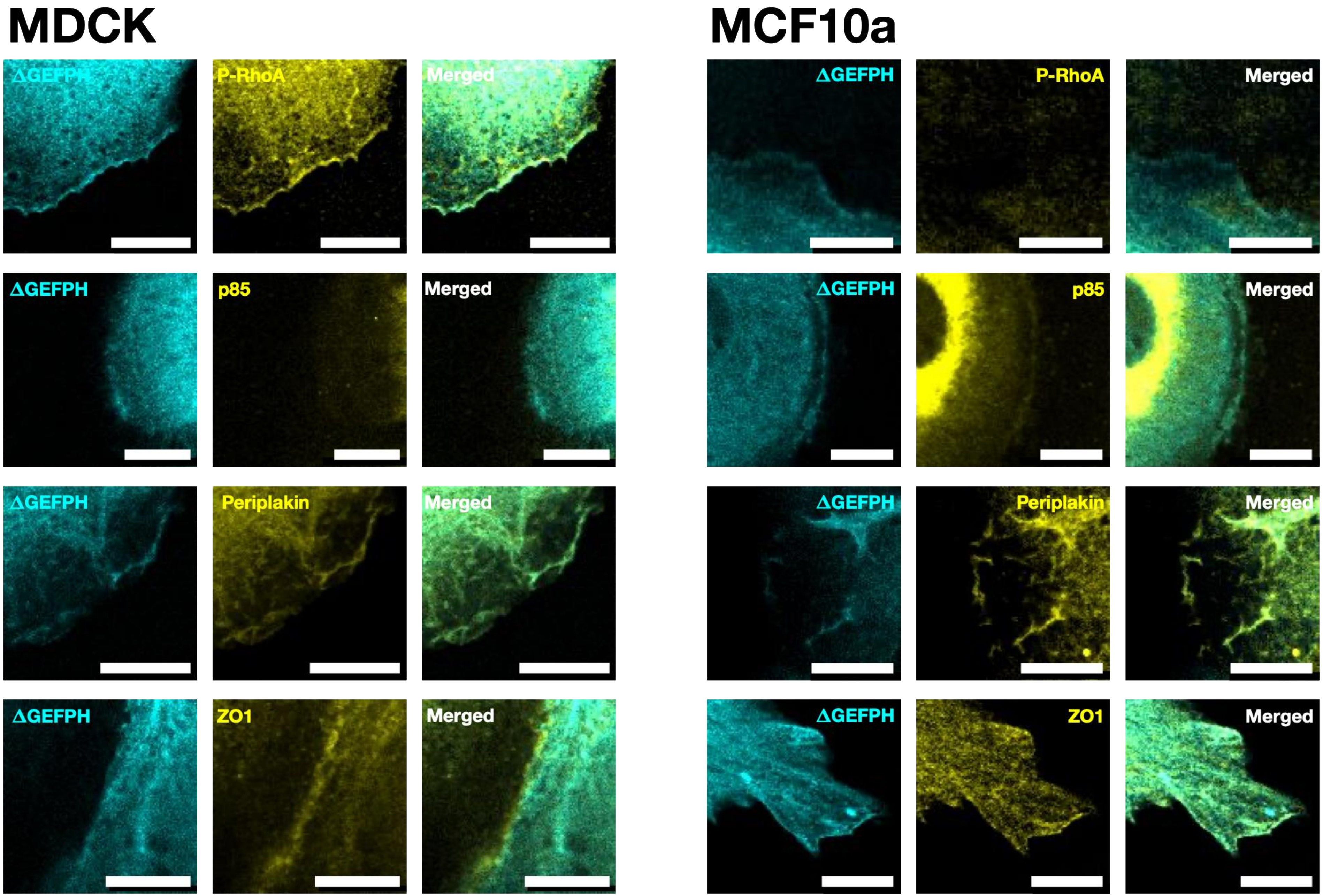

Supplemental Figure 9D

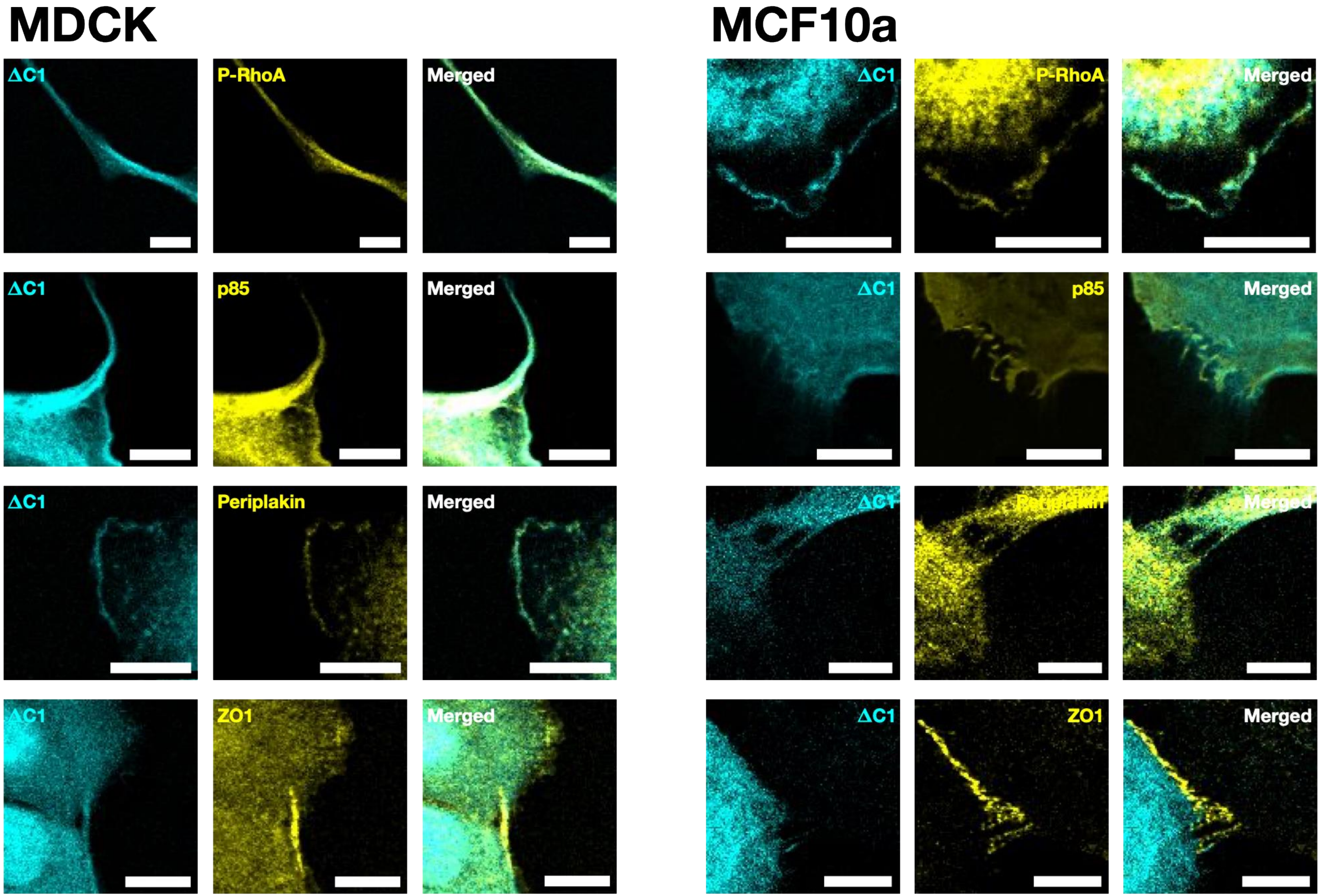

Supplemental Figure 9E

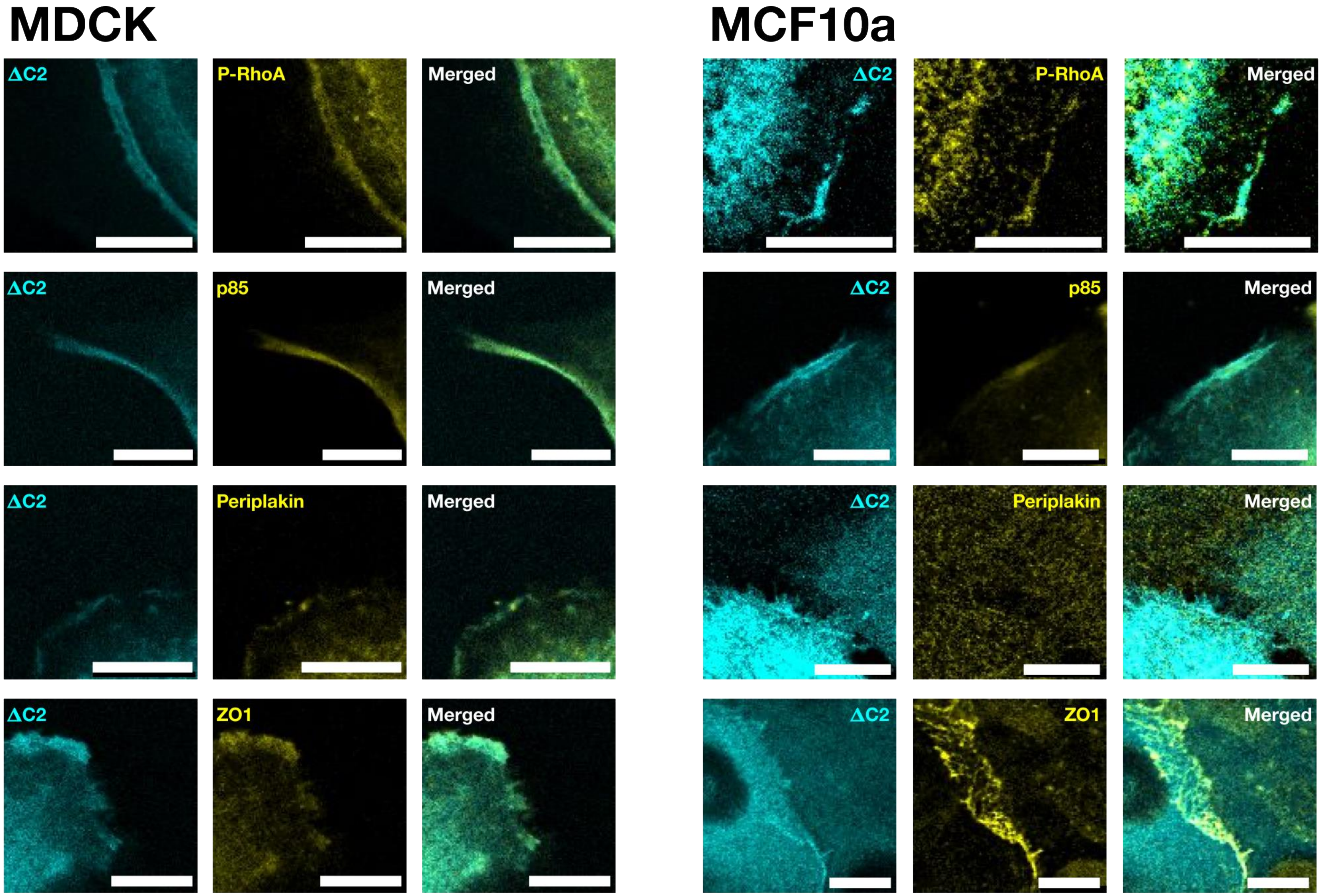

Supplemental Figure 9F

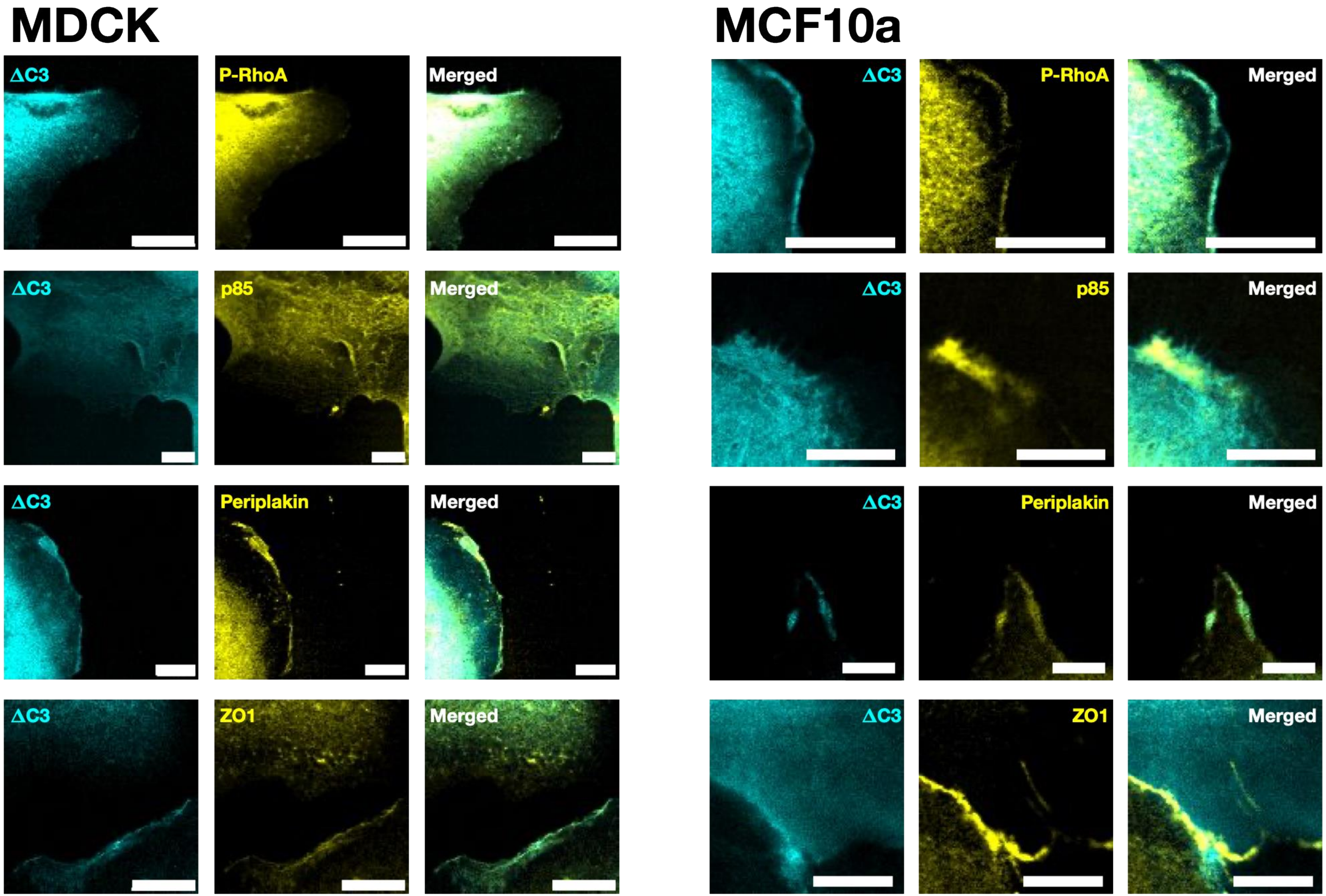

Supplemental Figure 10

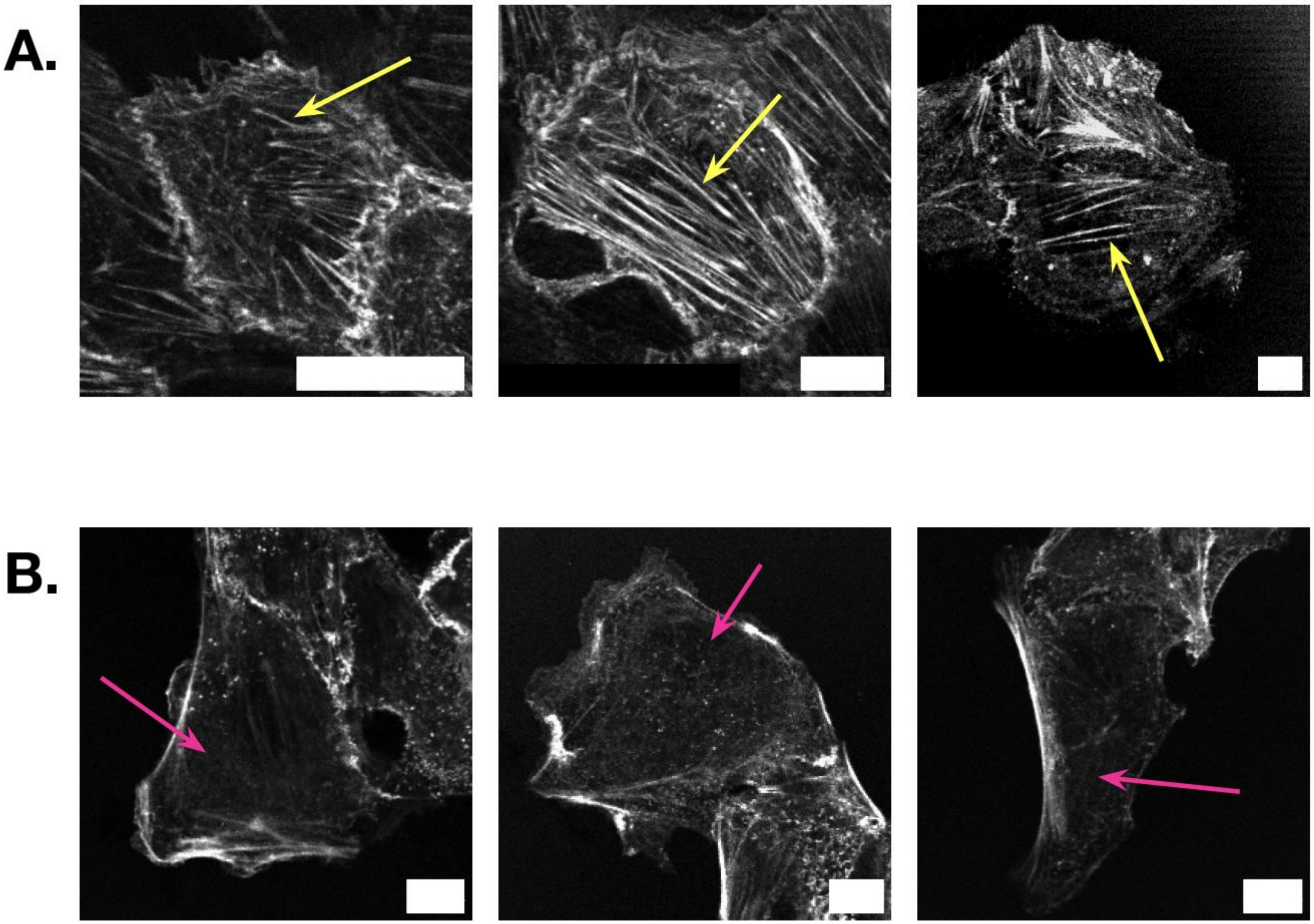

Supplemental Figure 11

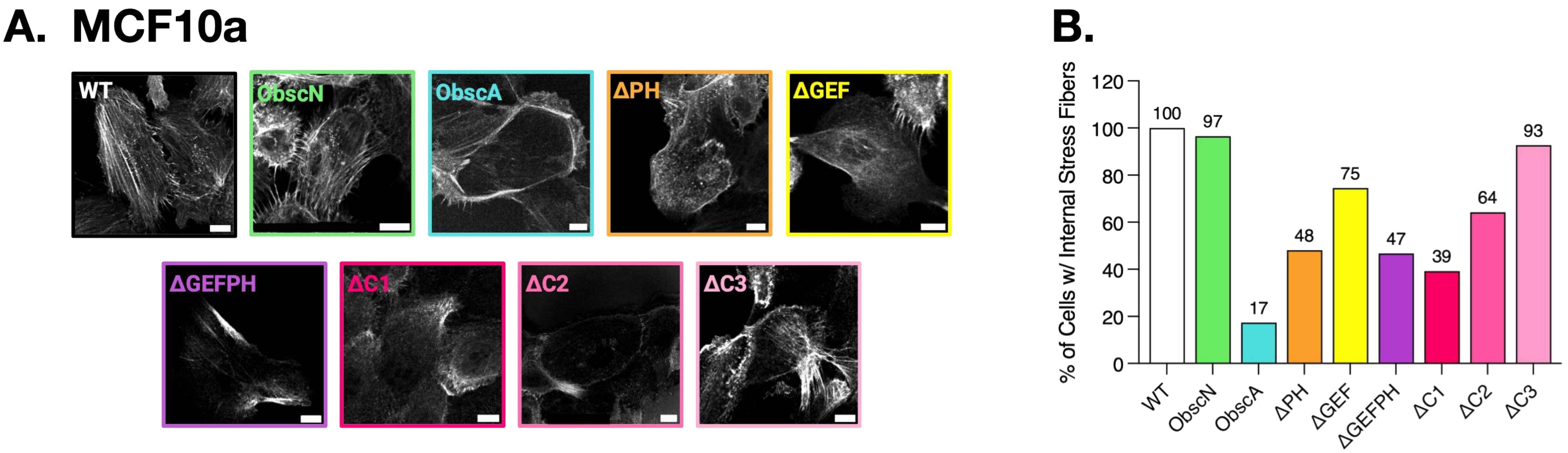

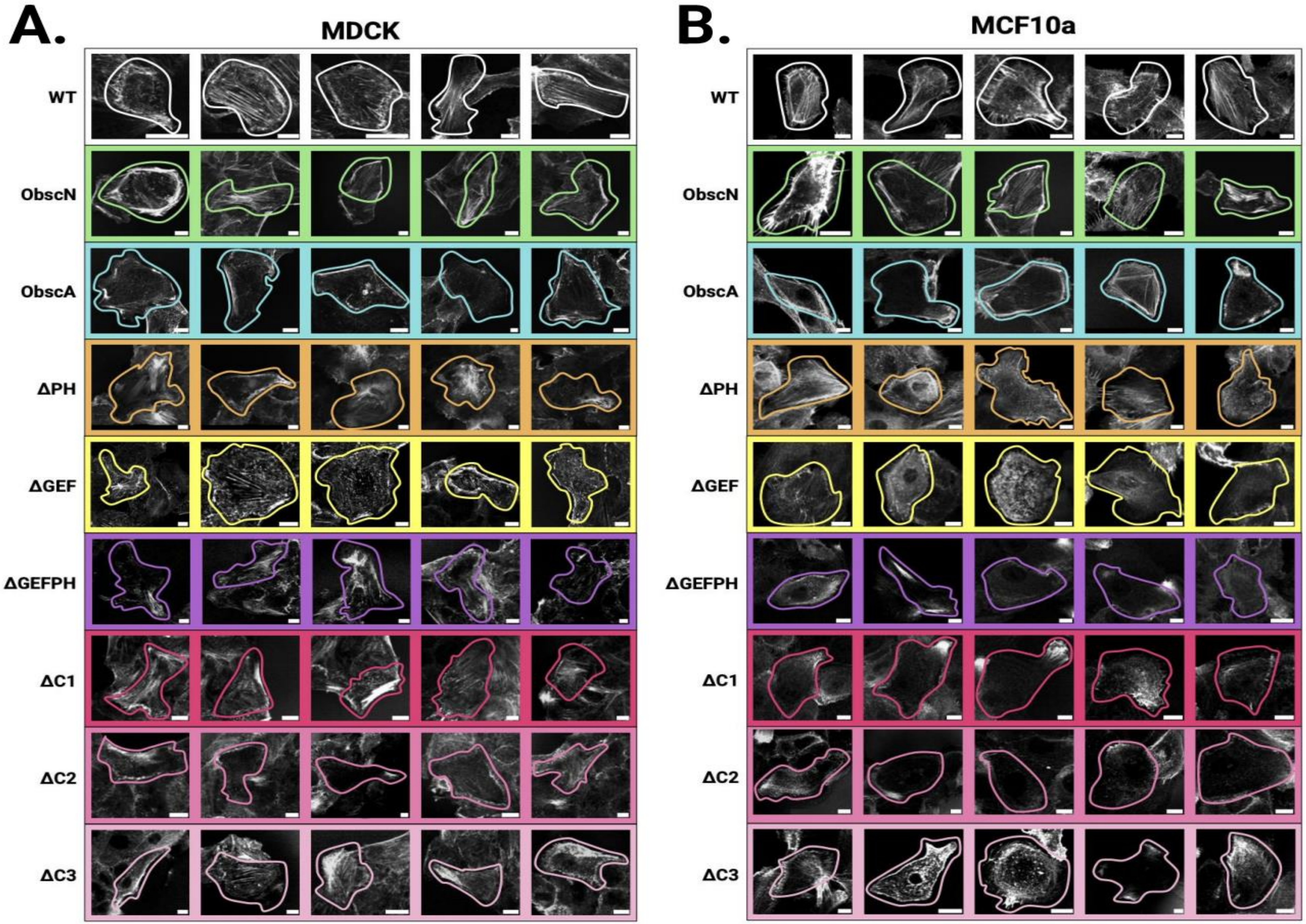

Supplemental Figure 12

Supplemental Figure 13

Normalized PIP3 Concentration in MCF10a cells	
**Treatment**	**PIP3 Concentration (pmol)**
**ObscN**	1.436 ± 0.1564
**ObscA**	1.075 ± 0.0378
**ΔPH**	1.104 ± 0.0377
**ΔGEF**	1.108 ± 0.0066
**ΔC3**	1.583 ± 0.0384
