# Supplementary material for: Obscurin a localizes near the cell membrane to modulate stress fiber dynamics and cell migration
Source: J Biol Chem. 2026 Mar 17;302(5):111383. doi: 10.1016/j.jbc.2026.111383 (PMC13100297; doi:10.1016/j.jbc.2026.111383)
Supplement: Supplementary material [file mmc2.docx]

Supplemental Figure legend:

**Supplemental Data 1.** Annotated BioID results for BirA-ObscA in MDCK cells

**Supplemental Data 2.** Annotated BioID results for BirA-ObscN in MDCK cells

**Supplemental Figure 1**. Cell line information.

**Supplemental Figure 2.** Primary and secondary antibody information.

**Supplemental Figure 3**. R code of a power analysis test to calculate the number of cells needed per treatment group to determine statistical significance between treatments.

**Supplemental Figure 4.** Workflow for cell migration analysis using mTrack2 and Trainable WEKA Segmentation plugins in ImageJ. The example of cell motion was previously published in [44].

**Supplemental Figure 5.** Colocalization coefficient values for ObscA with periplakin, ZO1, P85, and phosphorylated-RhoA.

**Supplemental Figure 6.** Blue Native gel/Western blots on whole cell MDCK lystates using α-mVenus antibodies show that both ObscA and ObscN run at the expected molecular weight of ~120 kDa. In addition, ObscA shows another band at around 240 kDa, suggesting the presence of dimer formation in these cells.

**Supplemental Figure 7**. Confocal images of mTFP-ObscA fluorescence in MDCK cells (A) and focal adhesion deficient MDCK ZO-1 KO cells (B) show a clear membrane localization in (A) and a lack of membrane localization in (B). (C) demonstrates fluorescenct Obsc-A in ZO-1 KO with membrane staining.

**Supplemental Figure 8.** Examples of membrane localization binning process. A) Purple arrows indicate distinct, clear obscurin membrane localization. B) Orange arrows indicate ambiguous or unclear obscurin membrane localization. All images were processed according to this binning pipeline. The white bar in the bottom right corner indicates 10μm.

**Supplemental Figure 9a**. ΔPH-infected MDCK and MCF-10A cells stained with various antibodies to detect colocalization. An established procedure using a 1:100 dilution of primary antibody and 1:500 dilution of either Goat anti-Mouse IgG (L + H) cross-adsorbed secondary antibody with conjugated AlexaFluor-647 (cat #A-21235, Ex/Em = 650/671) or Goat anti-Rabbit IgG (L + H) cross-adsorbed secondary antibody with conjugated AlexaFluor-647 (cat #A-21245, Ex/Em = 650/671). ΔPH was imaged using mTFP1 (Ex/Em = 462/492) and antibodies were imaged using AlexaFluor-647 (Ex/Em = 650/665) on the Leica Stellaris confocal microscope. The images selected are representative images for each treatment. Colocalization was quantified using Manders’ coefficients (tM1 and tM2), which were calculated using the Colocalization Threshold plugin in ImageJ. No background subtraction, imaging smoothing, or other preprocessing methods were used. Costes’ automated thresholding method was used to determine thresholds for each channel per treatment, with tM1 corresponding to the overlap of signals from channel 1 (mTFP1) to channel 2 (AlexaFluor-647), and tM2 corresponding to the overlap of signals from channel 2 (AlexaFluor-647) to channel 1 (mTFP1). ΔPH + Periplakin produced tM1/tM2 values of 0.9459/0.9057 in MDCK cells and 0.9609/0.7739 in MCF-10A cells, indicating strong colocalization consistent between cell types. ΔPH + ZO1 produced tM1/tM2 values of 0.9632/0.7929 in MDCK cells and 0.9911/0.9946 in MCF-10A cells, indicating strong colocalization consistent between cell types. ΔPH + P85 produced tM1/tM2 values of 0.9972/0.9843 in MDCK cells and 0.9826/0.9827 in MCF-10A cells, indicating strong colocalization consistent between cell types. ΔPH + P-RhoA produced tM1/tM2 values of 0.9598/0.9951 in MDCK cells and 0.8775/0.9373 in MCF-10A cells, indicating strong colocalization consistent between cell types.

**Supplemental Figure 9b**. ΔGEF-infected MDCK and MCF-10A cells stained with various antibodies to detect colocalization. An established procedure using a 1:100 dilution of primary antibody and 1:500 dilution of either Goat anti-Mouse IgG (L + H) cross-adsorbed secondary antibody with conjugated AlexaFluor-647 (cat #A-21235, Ex/Em = 650/671) or Goat anti-Rabbit IgG (L + H) cross-adsorbed secondary antibody with conjugated AlexaFluor-647 (cat #A-21245, Ex/Em = 650/671). ΔGEF was imaged using mTFP1 (Ex/Em = 462/492) and antibodies were imaged using AlexaFluor-647 (Ex/Em = 650/665) on the Leica Stellaris confocal microscope. The images selected are representative images for each treatment. Colocalization was quantified using Manders’ coefficients (tM1 and tM2), which were calculated using the Colocalization Threshold plugin in ImageJ. No background subtraction, imaging smoothing, or other preprocessing methods were used. Costes’ automated thresholding method was used to determine thresholds for each channel per treatment, with tM1 corresponding to the overlap of signals from channel 1 (mTFP1) to channel 2 (AlexaFluor-647), and tM2 corresponding to the overlap of signals from channel 2 (AlexaFluor-647) to channel 1 (mTFP1). ΔGEF + Periplakin produced tM1/tM2 values of 0.4426/0.3850 in MDCK cells and 0.9349/0.8989 in MCF-10A cells, indicating poor colocalization in MDCK cells and strong colocalization in MCF-10A cells.. ΔGEF + ZO1 produced tM1/tM2 values of 1.000/0.9991 in MDCK cells and 0.9931/0.9747 in MCF-10A cells, indicating strong colocalization consistent between cell types. ΔGEF + P85 produced tM1/tM2 values of 0.8499/0.8820 in MDCK cells and 0.9896/0.9716 in MCF-10A cells, indicating strong colocalization consistent between cell types. ΔGEF + P-RhoA produced tM1/tM2 values of 0.9497/0.9722 in MDCK cells and 0.9529/0.9950 in MCF-10A cells, indicating strong colocalization consistent between cell types.

**Supplemental Figure 9c**. ΔGEFPH-infected MDCK and MCF-10A cells stained with various antibodies to detect colocalization. An established procedure using a 1:100 dilution of primary antibody and 1:500 dilution of either Goat anti-Mouse IgG (L + H) cross-adsorbed secondary antibody with conjugated AlexaFluor-647 (cat #A-21235, Ex/Em = 650/671) or Goat anti-Rabbit IgG (L + H) cross-adsorbed secondary antibody with conjugated AlexaFluor-647 (cat #A-21245, Ex/Em = 650/671). ΔGEFPH was imaged using mTFP1 (Ex/Em = 462/492) and antibodies were imaged using AlexaFluor-647 (Ex/Em = 650/665) on the Leica Stellaris confocal microscope. The images selected are representative images for each treatment. Colocalization was quantified using Manders’ coefficients (tM1 and tM2), which were calculated using the Colocalization Threshold plugin in ImageJ. No background subtraction, imaging smoothing, or other preprocessing methods were used. Costes’ automated thresholding method was used to determine thresholds for each channel per treatment, with tM1 corresponding to the overlap of signals from channel 1 (mTFP1) to channel 2 (AlexaFluor-647), and tM2 corresponding to the overlap of signals from channel 2 (AlexaFluor-647) to channel 1 (mTFP1). ΔGEFPH + Periplakin produced tM1/tM2 values of 0.9989/0.9925 in MDCK cells and 0.8931/0.8771 in MCF-10A cells, indicating strong colocalization consistent between cell types. ΔGEFPH + ZO1 produced tM1/tM2 values of 0.5192/0.5911 in MDCK cells and 0.9653/0.9968 in MCF-10A cells, indicating moderate colocalization in MDCK cells and strong colocalization in MCF-10A cells. ΔGEFPH + P85 produced tM1/tM2 values of 0.6155.0.6473 in MDCK cells and 0.9813/0.7643 in MCF-10A cells, indicating moderate colocalization in MDCK cells and strong colocalization in MCF-10A cells. ΔGEFPH + P-RhoA produced tM1/tM2 values of 0.9023/0.9864 in MDCK cells and 0.4360/0.5480 in MCF-10A cells, indicating strong colocalization in MDCK cells and moderate colocalization in MCF-10A cells.

**Supplemental Figure 9d**. ΔC1-infected MDCK and MCF-10A cells stained with various antibodies to detect colocalization. An established procedure using a 1:100 dilution of primary antibody and 1:500 dilution of either Goat anti-Mouse IgG (L + H) cross-adsorbed secondary antibody with conjugated AlexaFluor-647 (cat #A-21235, Ex/Em = 650/671) or Goat anti-Rabbit IgG (L + H) cross-adsorbed secondary antibody with conjugated AlexaFluor-647 (cat #A-21245, Ex/Em = 650/671). ΔC1 was imaged using mTFP1 (Ex/Em = 462/492) and antibodies were imaged using AlexaFluor-647 (Ex/Em = 650/665) on the Leica Stellaris confocal microscope. The images selected are representative images for each treatment. Colocalization was quantified using Manders’ coefficients (tM1 and tM2), which were calculated using the Colocalization Threshold plugin in ImageJ. No background subtraction, imaging smoothing, or other preprocessing methods were used. Costes’ automated thresholding method was used to determine thresholds for each channel per treatment, with tM1 corresponding to the overlap of signals from channel 1 (mTFP1) to channel 2 (AlexaFluor-647), and tM2 corresponding to the overlap of signals from channel 2 (AlexaFluor-647) to channel 1 (mTFP1). ΔC1 + Periplakin produced tM1/tM2 values of 0.6433/0.9154 in MDCK cells and 0.6537/0.8186 in MCF-10A cells, indicating moderate colocalization consistent between cell types. ΔC1 + ZO1 produced tM1/tM2 values of 0.8806/0.7764 in MDCK cells and 0.1912/0.1997 in MCF-10A cells, indicating moderate colocalization in MDCK cells and very weak colocalization in MCF-10A cells. ΔC1 + P85 produced tM1/tM2 values of 0.9807/0.9995 in MDCK cells and 0.9971/0.9569 in MCF-10A cells, indicating strong colocalization consistent between cell types. ΔC1 + P-RhoA produced tM1/tM2 values of 0.8584/0.9995 in MDCK cells and 0.8372/0.8205 in MCF-10A cells, indicating moderate colocalization consistent between cell types.

**Supplemental Figure 9e**. ΔC2-infected MDCK and MCF-10A cells stained with various antibodies to detect colocalization. An established procedure using a 1:100 dilution of primary antibody and 1:500 dilution of either Goat anti-Mouse IgG (L + H) cross-adsorbed secondary antibody with conjugated AlexaFluor-647 (cat #A-21235, Ex/Em = 650/671) or Goat anti-Rabbit IgG (L + H) cross-adsorbed secondary antibody with conjugated AlexaFluor-647 (cat #A-21245, Ex/Em = 650/671). ΔC2 was imaged using mTFP1 (Ex/Em = 462/492) and antibodies were imaged using AlexaFluor-647 (Ex/Em = 650/665) on the Leica Stellaris confocal microscope. The images selected are representative images for each treatment. Colocalization was quantified using Manders’ coefficients (tM1 and tM2), which were calculated using the Colocalization Threshold plugin in ImageJ. No background subtraction, imaging smoothing, or other preprocessing methods were used. Costes’ automated thresholding method was used to determine thresholds for each channel per treatment, with tM1 corresponding to the overlap of signals from channel 1 (mTFP1) to channel 2 (AlexaFluor-647), and tM2 corresponding to the overlap of signals from channel 2 (AlexaFluor-647) to channel 1 (mTFP1). ΔC2 + Periplakin produced tM1/tM2 values of 0.7154/0.7444 in MDCK cells and 0.1881/0.0680 in MCF-10A cells, indicating moderate colocalization in MDCK cells and extremely weak colocalization in MCF-10A cells. ΔC2 + ZO1 produced tM1/tM2 values of 0.9530/0.9833 in MDCK cells and 0.2327/0.2994 in MCF-10A cells, indicating strong colocalization in MDCK cells and weak colocalization in MCF-10A cells. ΔC2 + P85 produced tM1/tM2 values of 1.000/0.9881 in MDCK cells and 0.8842/0.9883 in MCF-10A cells, indicating strong colocalization consistent between cell types. ΔC2 + P-RhoA produced tM1/tM2 values of 0.8046/0.8997 in MDCK cells and 0.6269/0.3553 in MCF-10A cells, indicating moderate colocalization consistent between cell types.

**Supplemental Figure 9f**. ΔC3-infected MDCK and MCF-10A cells stained with various antibodies to detect colocalization. An established procedure using a 1:100 dilution of primary antibody and 1:500 dilution of either Goat anti-Mouse IgG (L + H) cross-adsorbed secondary antibody with conjugated AlexaFluor-647 (cat #A-21235, Ex/Em = 650/671) or Goat anti-Rabbit IgG (L + H) cross-adsorbed secondary antibody with conjugated AlexaFluor-647 (cat #A-21245, Ex/Em = 650/671). ΔC3 was imaged using mTFP1 (Ex/Em = 462/492) and antibodies were imaged using AlexaFluor-647 (Ex/Em = 650/665) on the Leica Stellaris confocal microscope. The images selected are representative images for each treatment. Colocalization was quantified using Manders’ coefficients (tM1 and tM2), which were calculated using the Colocalization Threshold plugin in ImageJ. No background subtraction, imaging smoothing, or other preprocessing methods were used. Costes’ automated thresholding method was used to determine thresholds for each channel per treatment, with tM1 corresponding to the overlap of signals from channel 1 (mTFP1) to channel 2 (AlexaFluor-647), and tM2 corresponding to the overlap of signals from channel 2 (AlexaFluor-647) to channel 1 (mTFP1). ΔC3 + Periplakin produced tM1/tM2 values of 0.8354/0.9972 in MDCK cells and 0.9722/0.7101 in MCF-10A cells, indicating strong colocalization consistent between cell types. ΔC3 + ZO1 produced tM1/tM2 values of 0.9563/0.9913 in MDCK cells and 0.6257/0.7488 in MCF-10A cells, indicating strong colocalization in MDCK cells and moderate colocalization in MCF-10A cells. ΔC3 + P85 produced tM1/tM2 values of 0.7952/0.7044 in MDCK cells and 0.7538/0.7359 in MCF-10A cells, indicating moderate colocalization consistent between cell types. ΔC3 + P-RhoA produced tM1/tM2 values of 0.9911/0.9946 in MDCK cells and 0.7412/0.9990 in MCF-10A cells, indicating strong colocalization consistent between cell types.

**Supplemental Figure 10.** Examples of actin fiber binning process. These data are also shown in Figure 4A, since these data best demonstrate the actin phenotype. A) yellow arrows indicate distinct, clear central actin fiber localization. B) pink arrows indicate ambiguous or absent central actin fiber localization. All images were processed according to this binning pipeline. The white bar in the bottom right corner indicates 10um.

**Supplemental Figure 11.** Stress fibers present in MCF-10A cells. Phalloidin staining in MCF-10A cells at a 1:500 dilution for WT, ObscN, and ObscA, ΔPH, ΔGEF, ΔGEFPH, ΔC1, ΔC2, and ΔC3 treatments. Cells infected with various obscurin constructs were incubated for 24-48 hours prior to imaging on the Leica Stellaris SP8 confocal microscope. The Plan Fluor 63x/1.40NA objective with oil immersion and a 0.18µm resolution were used for fluorescence microscopy. Obscurin was visualized using the mTFP1 (Ex/Em = 462/492) channel actin stress fibers were visualized using the AlexaFluor-594 (Ex/Em = 590/618) channel, and an overlay of the two channels was generated. The mTFP1 channel laser line was at 462nm and the detection range was 487-571nm. The AlexaFluor-594 channel laser line was at 590nm and the detection range was 595-834nm. The pixel range of all images was 0.19-0.48µm. Single images of both channels were generated simultaneously, with 1 Airy Unit pinhole size, and were 8-bit. A minimum of 25 cells per treatment were collected for data analysis **A)** Representative images for each treatment in MCF-10A cells. Images were zoomed in 1.5-2x through ImageJ and the scale bar reflects the changes. **B)** Total approximate percentage of stress fibers present in WT (n = 57), ObscN (n = 25), ObscA (n = 30), ΔPH (n = 41), ΔGEF (n = 25), ΔGEFPH (n = 32), ΔC1 (n = 32), ΔC2 (n = 35), and ΔC3 (n = 31) in MCF-10A cells. The presence of any stress fibers in the central or peripheral areas of a cell were classified as having stress fibers.

**Supplemental Figure 12.** Representative Phalloidin staining of various obscurin treatments. WT and ObscA-, ObscN-, ΔPH-, ΔGEF-, ΔC1-, ΔC2-, ΔC3-infected MDCK (**A**) and MCF-10A (**B**) cells stained with Phalloidin-594 at a 1:500 dilution. Plates infected with obscurin were incubated for 24-48 hours prior to staining and imaging on the Leica Stellaris SP8 confocal microscope. **A**) 5 representative MDCK cells per treatment were selected to display stress fiber phenotypes. **B**) 5 representative MCF-10A cells per treatment were selected to display stress fiber phenotypes. Not all cells in images that contain more than one cell contained obscurin excluding WT, so the representative cell that was outlined is the cell of interest. The outlines were made based on the obscurin localization present in the mTFP1 channel. The Plan Fluor 63x/1.40NA objective with oil immersion and a 0.18µm resolution were used for fluorescence microscopy. Obscurin was visualized using the mTFP1 (Ex/Em = 462/492) channel actin stress fibers were visualized using the AlexaFluor-594 (Ex/Em = 590/618) channel, and an overlay of the two channels was generated. The mTFP1 channel laser line was at 462nm and the detection range was 487-571nm. The AlexaFluor-594 channel laser line was at 590nm and the detection range was 595-834nm. The pixel range of all images was 0.19-0.48µm. Single images of both channels were generated simultaneously, with 1 Airy Unit pinhole size, and were 8-bit. Cells selected for imaging were required to have obscurin present (excluding WT) and clear stress fibers or other actin-related structures. Images were exported to ImageJ where pre-processing and analysis was conducted. Images were zoomed in 1.5-2x with a 10µm scale bar adjusted accordingly. No additional changes to brightness or contrast were made.

**Supplemental Figure 13.** Normalized PIP_3_ concentration in MCF10A cells, in pmol/dish
